# Stereoselective Remote Functionalization via Palladium‐Catalyzed Redox‐Relay Heck Methodologies

**DOI:** 10.1002/chem.202002849

**Published:** 2020-10-08

**Authors:** Holly E. Bonfield, Damien Valette, David M. Lindsay, Marc Reid

**Affiliations:** ^1^ Chemical Development GlaxoSmithKline Gunnels Wood Road Stevenage Hertfordshire SG1 2NY UK; ^2^ Department of Pure and Applied Chemistry WestCHEM University of Strathclyde 295 Cathedral Street Glasgow Scotland G1 1XL UK

**Keywords:** asymmetric catalysis, chain walking, Heck reaction, palladium, relay

## Abstract

Exploration of novel, three‐dimensional chemical space is of growing interest in the drug discovery community and with this comes the challenge for synthetic chemists to devise new stereoselective methods to introduce chirality in a rapid and efficient manner. This Minireview provides a timely summary of the development of palladium‐catalyzed asymmetric redox‐relay Heck‐type processes. These reactions represent an important class of transformation for the selective introduction of remote stereocenters, and have risen to prominence over the past decade. Within this Minireview, the vast scope of these transformations will be showcased, alongside applications to pharmaceutically relevant chiral building blocks and drug substances. To complement this overview, a mechanistic summary and discussion of the current limitations of the transformation are presented, followed by an outlook on future areas of investigation.

## Graphical abstract

**Palladium walks the walk**: This Minireview highlights the stereoselective synthesis of remote tertiary and quaternary stereogenic centers by a palladium‐catalyzed redox‐relay Heck strategy. Over the past decade, expansion of this methodology has enabled the creation of a diverse set of substrates of interest to the pharmaceutical industry (see scheme).

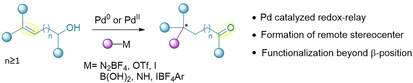

## 1 Introduction

Over the past two decades, the over‐reliance on a limited set of reactions in drug discovery has led to chemical libraries that are densely populated with molecules rich in sp^2^ character.^[1]^ In recent years however, increased interest in expanding chemical space, particularly the exploration of three‐dimensional structures, has been fueled by the evolution of synthetic chemistry methodology in the pharmaceutical industry via the implementation of high throughput experimentation and directed evolution screening platforms.^[2]^ Increasing the fraction of sp^3^ carbons is appealing from a structural diversity perspective because a higher degree of three‐dimensionality provides access to new target classes. From an industrial standpoint, such approaches can lead to a competitive advantage.^[1]^ Incorporation of a stereocenter offers multiple benefits, such as enhanced solubility, pharmacokinetics and selectivity by providing more complementary binding to the active sites of proteins.[^2a^, ^3^] This observation is supported by the greater proportion of chiral molecules that progress in clinical trials.^[4]^

Stereoselectivity is a major theme in the discovery, development and launch of new drugs because chiral drug molecules are almost exclusively marketed as single enantiomers.^[5]^ In addition, development and sales of chiral drugs continue to grow.^[6]^ As a result, there has been an increased interest in developing robust, cost‐efficient stereoselective processes for the large scale synthesis of active pharmaceutical ingredients (APIs) containing one or more stereogenic centers.^[7]^ Despite these efforts, major challenges remain. For instance, few, if any, drug molecules currently on the market have quaternary stereocenters installed via chemical synthesis.[^2a^, ^8^] Consequently, the development of broad, practical methodologies which can provide access to novel chiral motifs that are useful for drug design, with the added potential to streamline the synthesis of APIs, are of high value to industry and the synthetic community in general.^[9]^

### 1.1 Synthesis of remote stereogenic centers

Asymmetric catalysis is a long‐standing field of organic synthesis.^[10]^ The stereoselective α‐ and β‐functionalization of carbonyls has been extensively studied,^[11]^ but significantly less attention has been directed at the stereoselective synthesis of remote tertiary and quaternary stereocenters.^[12]^

**Scheme 1:**
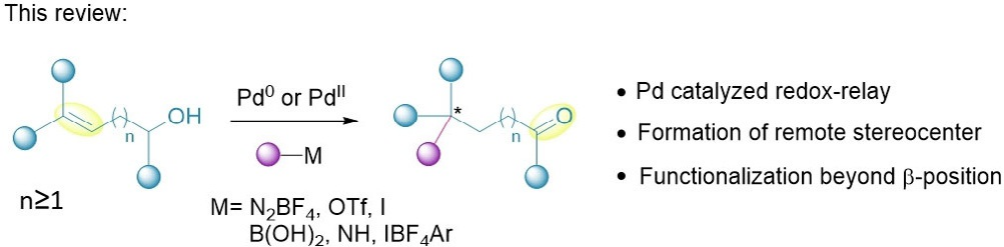
Scope of redox‐relay Heck‐type transformations covered in this Minireview.

This Minireview describes the development of palladium‐catalyzed asymmetric Heck‐type transformations. Other transition metals can also be applied, but these complementary methods are beyond the scope of the present article. Specifically, this review focuses on stereoselective synthetic transformations generating remote tertiary and quaternary stereogenic centers via a redox‐relay mechanism (Scheme 1). The two‐step sequence of this methodology, installation of a remote group with high stereoselectivity and subsequent oxidation of an alcohol linked by the redox‐relay mechanism, enables non‐trivial retrosynthetic disconnections to be considered. Herein, *remote functionalization* is defined as functionalization beyond the β‐position (*γ*, *δ*, *ϵ* etc.), and a *relay* is defined as migration over more than one bond. However, where appropriate, seminal examples not fulfilling the aforementioned criteria have been included to provide context.^[13]^

### 1.2 Redox‐relay chain walking systems

**Scheme 2:**
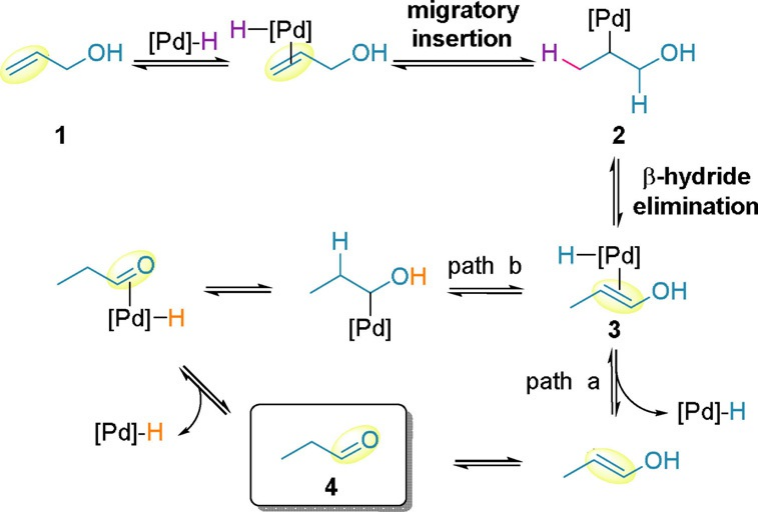
Palladium‐catalyzed chain walking mechanism in allylic alcohol systems.

Alkene migration using palladium as a catalyst has been known since 1926.^[14]^ This methodology, also known as *chain walking*, is common in polymer chemistry,^[15]^ and is now becoming more prevalent in small molecule synthesis.^[16]^ Of interest to the topic of this review is the use of palladium catalysts to facilitate alkene migration in allylic alcohol systems to generate carbonyls in a single step, thereby avoiding the need for further oxidation state manipulations.^[17]^ Such a transformation, also referred to as a *redox‐relay* event, formally repositions the unsaturation from an alkene to an alcohol. A general chain walking mechanism of allylic alcohol **1** is summarized in Scheme 2.^[17]^ An initial migratory insertion of the alkene into the palladium hydride bond yields **2**. Subsequent β‐hydride elimination leads to enol **3**, which can either tautomerize (path a), or undergo a migratory insertion/oxidative deprotonation sequence (path b). During this final step, the alcohol redox acceptor is converted to the corresponding carbonyl product **4**, which serves as a thermodynamic sink.

**Scheme 3:**
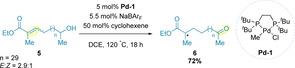
Palladium‐catalyzed redox‐relay.

Alkene migration has been shown to operate over a considerable number of C−C bonds, in good yield, and with a broad substrate scope. Of particular interest, Mazet and co‐workers have demonstrated migrations along alkyl chains where the alkene ‘deconjugates’ from a carbonyl group using catalyst **Pd‐1**.^[18]^ The palladium hydride species required for the transformation is generated in situ via halide abstraction using an equivalent amount (with respect to the catalyst) of sodium tetrakis(3,5‐bis(trifluoromethyl)‐phenyl)borate) (NaBAr_F_) in the presence of cyclohexene, which serves as a sacrificial alkene additive.^[18a]^ Under such conditions, an impressive migration of the alkene in substrate **5**, along an alkyl chain 30 carbons in length, is demonstrated, yielding dicarbonyl **6** in 72 % yield (Scheme 3).^[18b]^

Mazet's redox‐relay work has also been extended to allow this migration to occur enantioselectively using **Pd‐2**, containing a chiral, bidentate phosphine ligand ((*R*,*R*)‐*i*Pr‐DUPHOS), electronically similar to **Pd‐1**. The reaction is stereospecific, with (*E*)‐ and (*Z*)‐alkenes (**7**) reported to generate the (*S*)‐ and (*R*)‐aldehyde products (**8**), respectively (Table 1).^[18a]^

**Table 1:** Enantioselective alkene migration with phosphine ligands.

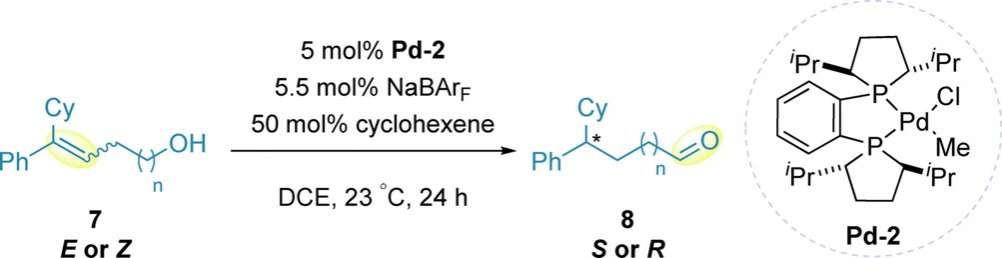					
Entry	*E*/*Z*	n	*R*/*S*	Yield [%]	e.e. [%]
1	*E*	0	*S*	65	73
2	*Z*	0	*R*	50	48
3	*E*	1	*S*	36	62

**Scheme 4:**
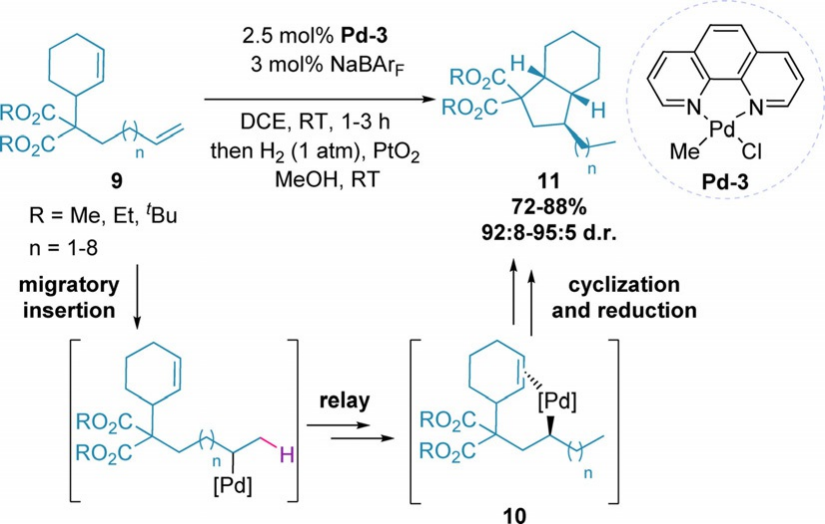
Enantioselective alkene migration with *N*,*N*‐bidentate ligands.

Similar methodologies have taken advantage of the aforementioned isomerization to enable intramolecular cyclizations over extended C−C chains.[^16a^, ^16b^, ^16f^, ^19^] Of note, Kochi and co‐workers demonstrated an approach to access bicyclo[4.3.0]nonane derivatives (**11**) in a stereoselective manner using **Pd‐3**, coordinated by an *N*,*N*‐bidentate ligand (Scheme 4).^[20]^ The most sterically accessible terminal alkene in **9** initiates the chain walking process, which terminates when the palladium is able to complex to the second alkene (**10**) and undergo a 5‐*exo*‐*trig* carbopalladation, which, after reduction, gives product **11**.

### 1.3 Heck reaction—Seminal redox‐relay studies

The C−C bond forming Heck, or Mizoroki–Heck, reaction is a palladium‐catalyzed coupling of an unsaturated halide (or triflate) with an alkene to generate a substituted alkene.^[21]^ The reaction was independently discovered by Mizoroki and Heck, and their co‐workers, in 1971 and 1972, respectively.^[22]^ Since these initial reports, the reaction has undergone considerable development, and today represents one of the foremost C−C coupling processes applied in organic synthesis. This reaction class has proven useful due to the mild conditions employed, together with the tolerance of a broad range of functional groups.^[23]^

**Scheme 5:**
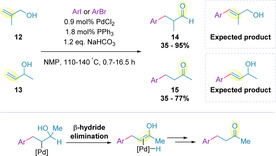
Unexpected chain walking products observed when exploring the scope of the Heck reaction.

During early investigation into the scope of this reaction, the formation of unexpected products was observed when using allylic alcohols as the alkene partner. Specifically, arylation of primary‐ and secondary‐allylic alcohols (**12** and **13**) resulted in the formation of 3‐aryl aldehydes (**14**) and ketones (**15**), respectively (Scheme 5).^[24]^ Primary‐ and secondary‐homoallylic alcohols were subsequently examined, and these also resulted in the formation of the corresponding aldehyde and ketone products, albeit in lower yield.[^24a^, ^24b^, ^25^] It was hypothesized that a ‘chain walking’ mechanism may be occurring, where the double bond migrates along the alkyl chain by iterative β‐hydride elimination/migratory insertion steps, until captured by the alcohol moiety to form the carbonyl. In support of this proposal, the propensity for palladium migration in other Heck‐type transformations has been well‐documented.^[26]^

Further optimization of the chain walking methodology broadened the scope of the reaction so that various unsaturated alcohols afforded the corresponding aldehyde/ketone, regardless of how remotely the center of unsaturation was located away from the hydroxyl group.^[27]^ In 1987, studies into a possible enantioselective variant of the reaction were conducted using chiral allylic alcohols,^[28]^ and concluded that the extent of chirality transfer was limited under the conditions employed.^[29]^

## 2 The Redox‐Relay Heck Reaction

### 2.1 Preliminary work

**Scheme 6:**
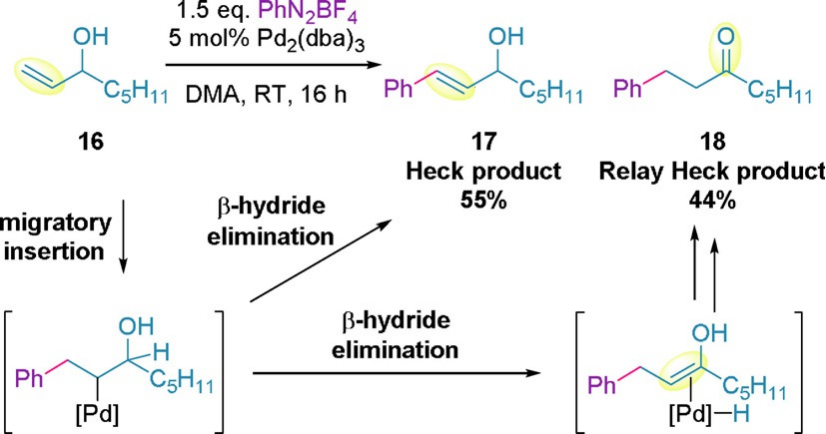
Preliminary work on the Heck reaction.

From 2010, Sigman and co‐workers began investigations into Heck transformations with the aim of developing a general, operationally simple and highly regioselective set of reaction conditions for the arylation of electronically non‐biased alkenes.^[30]^ Applying the group‘s previously optimized reaction conditions on allylic alcohol **16**, and using aryl diazonium salts as coupling partners, an approximately 1:1 ratio of the styrenyl Heck product **17** and ketone **18** was obtained, with the latter referred to as the *relay Heck* product (Scheme 6).[^30b^, ^31^]

Sigman's finding, coupled with the fact that limited success in an enantioselective variant of this transformation had been achieved thus far, prompted further investigation into the reaction. The authors hypothesized that a regio‐ and enantio‐selective transformation could be achieved by careful selection of a chiral catalyst that could electronically differentiate between the C−H bonds leading down the two relay pathways, whilst also sterically differentiating the two faces of the alkene.^[31]^

### 2.2 Arylation

**Figure 1:**
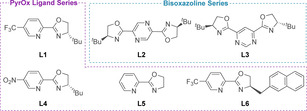
PyrOx and bisoxazoline ligand series discussed in this Minireview.

**Scheme 7:**
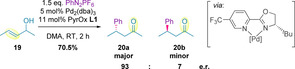
Enantioselective redox‐relay Heck reaction and coordination mode of PyrOx **L1**.

Since the redox‐relay Heck reaction was thought to proceed via successive β‐hydride elimination/migratory insertion steps, following an initial migratory insertion of the aryl‐metal complex into the alkene, it was postulated that an electrophilic catalyst would promote binding of the catalyst to the alkene of the substrate over competing dissociation.^[31]^ It was also noted that aryl diazonium salts may be incompatible with commonly used chiral phosphines, and so alternative chiral ligands were investigated.[^30^, ^31^, ^32^] The arylation of allylic alcohol **19** in the presence of pyridine‐oxazoline (PyrOx) ligand **L1** (Figure 1) was found to generate the corresponding ketone **20** in 93:7 e.r. (Scheme 7). The high enantioselectivity achieved was attributed to the chelating ligand generating a well‐defined environment for asymmetric induction.^[31]^

Since this initial publication, a range of empirically‐derived PyrOx and related pyrazine and pyrimidine bisoxazoline ligands have facilitated the vast expansion of this methodology (Figure 1).^[33]^ More recently, novel, computationally‐derived additions to the ligand series have been derived using multivariate regression analyses to build predictive correlation models based on substrate and catalyst parameterization.[^33^, ^34^] A combination of electronic (natural bond orbital, NBO, charges, and IR frequencies and intensities) and steric (Sterimol and Buried Volume) descriptors have been used to this end. It has been found that a more negative NBO charge on the oxazoline nitrogen atom of the PyrOx ligand correlates with increased enantioselectivities and the regioselectivity of arylation across the alkene is strongly correlated with all steric dimensions of the oxazoline 4‐substituent, as quantified by Sterimol and Buried Volume.

Subsequent investigation into the generation of remote tertiary stereocenters demonstrated that higher enantioselectivities were generally achieved when bulkier or more branched substituents on the saturated side of the allylic alcohol were used (Entry 1 vs. 2 in Table 2).^[31]^ It was also observed that when (*Z*)‐alkenes were subjected to the same conditions, the reaction proceeded in high enantioselectivity to give the antipodal ketone product (Entries 2 and 3, and 4 and 5). In all cases, racemic allylic alcohols (**21**) afforded the corresponding ketones (**22 a**/**b**) in high enantioselectivity, strongly suggesting catalyst control over the stereochemical outcome of the transformation. The reaction was also selective with primary and secondary homoallylic‐ and *bis*‐homoallylic‐alcohols, in terms of both enantio‐ and regio‐selectivity (Entries 6–9), favoring the regioisomer resulting from C−C bond formation at the distal alkene carbon relative to the alcohol. The effect of distance between the alcohol and the alkene in the substrate, termed the *chain length*, was also examined. Whilst enantioselectivities remained high, diminished yields were observed over longer chain lengths (Compare Entries 1, 7 and 9).

**Table 2:** Substrate scope for the enantioselective redox‐relay Heck arylation.

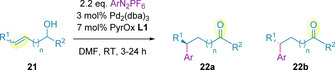							
Entry	*E*/*Z*	*n*	R^1^	R^2^	Ar	Yield [%]	**a/b^[a]^**
1	*E*	0	Me	Me	*p‐*COMePh	65	91:9
2	*E*	0	Me	C_8_H_17_	*p‐*COMePh	68	96:4
3	*Z*	0	Me	C_8_H_17_	*p‐*COMePh	75	4:96
4	*Z*	0	Me	^*i*^Pr	*p‐*NO_2_Ph	77	3:97
5	*E*	0	Me	^*i*^Pr	*p‐*NO_2_Ph	59	95:5
6	*Z*	1	Et	Me	*p‐*CO_2_MePh	79	4:96
7	*E*	1	Et	Me	*p‐*CO_2_MePh	58	90:10
8	*Z*	1	Et	H	*p‐*CO_2_MePh	63	2:98
9	*E*	2	Me	Me	*p‐*CO_2_MePh	52	97:3

[a] 4:1 site selectivity for *δ*‐ versus *γ*‐insertion.

**Scheme 8:**
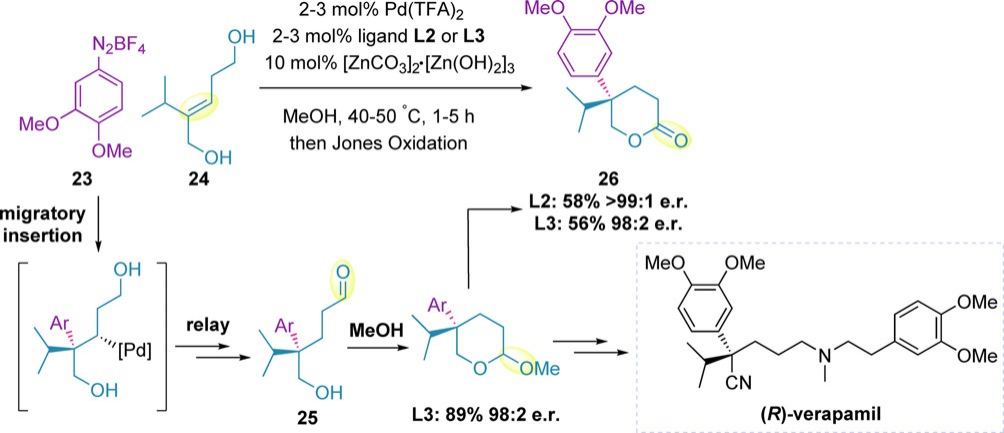
Redox‐relay Heck γ‐lactone quaternary stereocenter generation toward the synthesis of (*R*)‐verapamil.

Correia and co‐workers have also utilized aryl diazonium salts in the redox‐relay Heck methodology to generate quaternary stereocenters. The authors reported that the use of tetrafluoroborate diazonium salts (**23**) enabled operationally simplified conditions to be developed, compared to the corresponding hexafluorophosphates, for the efficient arylation of (*Z*)‐diols (**24**).^[35]^ In this sequence, the resulting aldehyde (**25**), reacts intramolecularly to generate lactone **26** (after oxidation). The substrate scope primarily consisted of β‐functionalized lactones, with a single example of *γ*‐lactone formation (Scheme 8),^[35b]^ and all proceeded with excellent enantioselectivities. Subsequently, these general redox‐relay Heck conditions have been successfully transferred to a flow set‐up.^[36]^

The abovementioned diazonium‐centered methodology was also showcased in a 6‐step stereoselective synthesis of (*R*)‐verapamil, a calcium channel blocker currently commercialized as a racemate,^[37]^ in 29 % overall yield.^[35b]^ The redox‐relay Heck reaction was used as the key step and proceeded in excellent yield and enantioselectivity (Scheme 8).

**Scheme 9:**
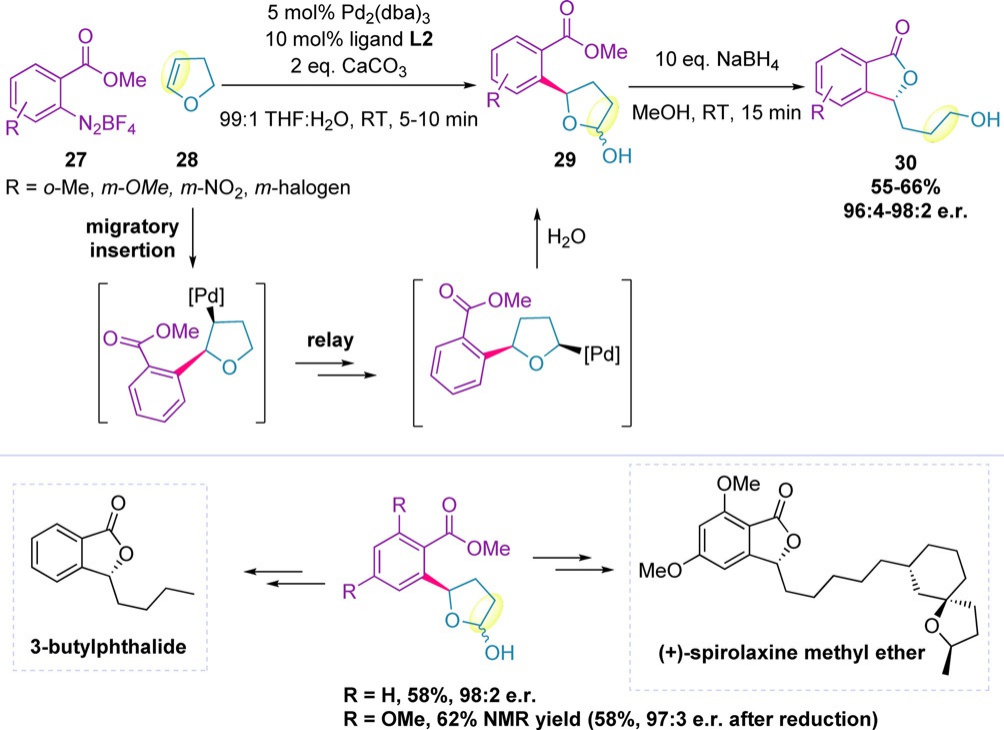
Phthalide synthesis and applications.

The arylation of 2,3‐dihydrofuran **28** to furnish lactols (**29**), by nucleophilic attack of water following migration, was subsequently established (Scheme 9).^[38]^ Following reduction, phthalides (**30**) were obtained in high enantiomeric ratios and in 55–66 % yield over the two steps. The synthetic utility of the methodology was showcased in both the total synthesis of the natural product 3‐butylphthalide, and the formal synthesis of an advanced intermediate towards (+)‐spirolaxine methyl ether, an anti‐Heliobacter pyroli agent.^[38]^

### 2.3 Alkenylation

**Scheme 10:**
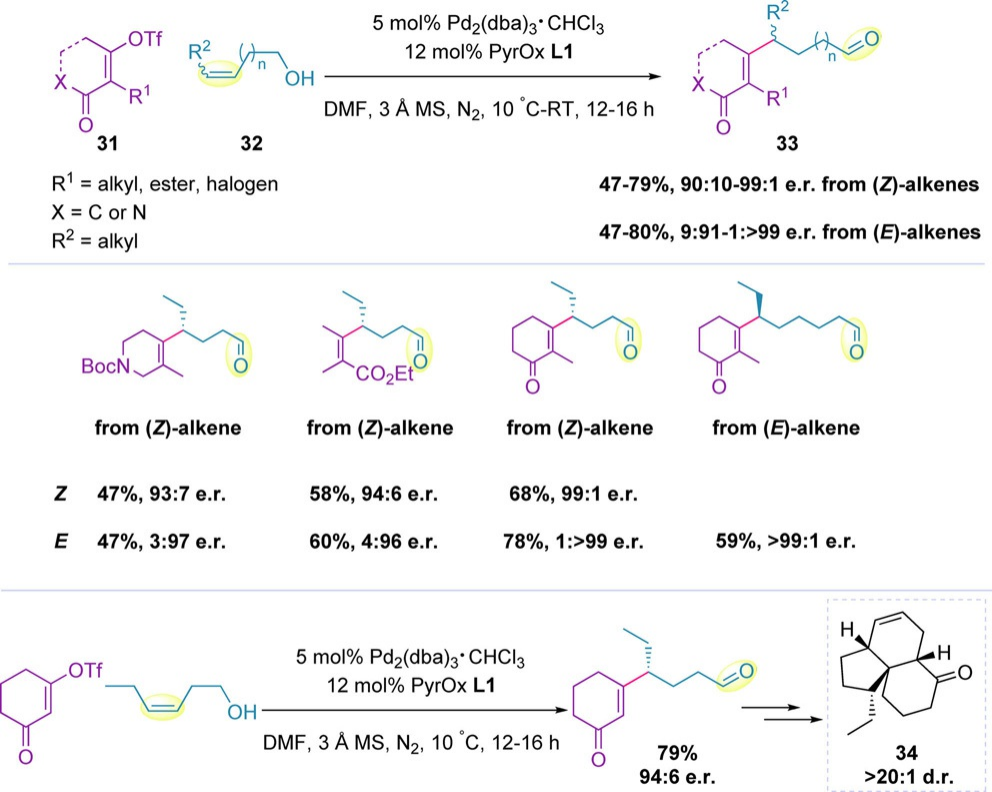
Redox‐relay Heck alkenylation of disubstituted alkenols.

The electron‐deficient nature of alkenyl triflates (**31**) was proposed by Sigman and co‐workers to favor the desired redox‐relay mechanism, achieving site‐selective β‐hydride elimination following migratory insertion.^[39]^ A powerful protocol using PyrOx **L1** was developed for the selective addition to disubstituted alkenols (**32**) (Scheme 10). Both (*E*)‐ and (*Z*)‐alkenols can be used in the reaction, with the different alkene geometries leading to opposite enantiomers as the major product. In all cases, high enantiomeric ratios were attained irrespective of the electronic nature of the substrate or the chain length.^[39]^ The redox‐relay Heck alkenylation of alkenols has also been demonstrated in the synthesis of chiral building block **34**, present in bioactive molecules such as elisabethin A, elisapterosin B and colombiasin A.^[39]^

Application of the alkenylation methodology to trisubstituted alkenols (**36**) was initially unsuccessful.^[40]^ It was hypothesized that the greater steric demand of the trisubstituted alkene was impeding the migratory insertion of the electron‐deficient alkenyl triflate. Evaluation of electron‐rich alkenyl triflates revealed that a more electrophilic ligand (**L4**, Figure 1) was required to enhance binding of the chiral ligand and substrate, which in turn increased the rate of migratory insertion (Table 3).^[40]^ In general, high enantioselectivities were achieved, regardless of the electronic nature of the alkenyl triflate (**35**). In contrast to the disubstituted alkenol systems, only (*E*)‐alkenyl triflates can be used in the trisubstituted system, which the authors suggest indicates a sterically governed migratory insertion. For more sterically demanding trisubstituted alkenols, higher catalyst loadings and longer reaction times were required. Furthermore, the yield was found to decrease with increased migration distance when an additional methylene unit was added (Entry 1 vs. Entry 2).

**Table 3:** Alkenylation of trisubstituted alkenols to generate quaternary stereocenters.

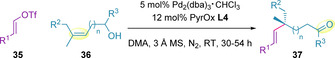						
Entry	R^1^	R^2^	R^3^	*n*	Yield[%]	e.r.
1	Ph	Me	H	1	81	95:5^[a]^
2	Ph	Me	H	2	35	93:7^[a]^
3	Ph	^*n*^Pr	H	1	74	95:5^[a]^
					72	13:87^[b]^
4	Cy	Me	H	1	75	97:3^[a]^
5	C_5_H_11_	Me	H	1	55	96:4^[a]^
6	C_5_H_11_	OTBS	Me	2	33	87:13^[b]^

[a] From (*Z*)‐alkene. [b] From (*E*)‐alkene.

**Scheme 11:**
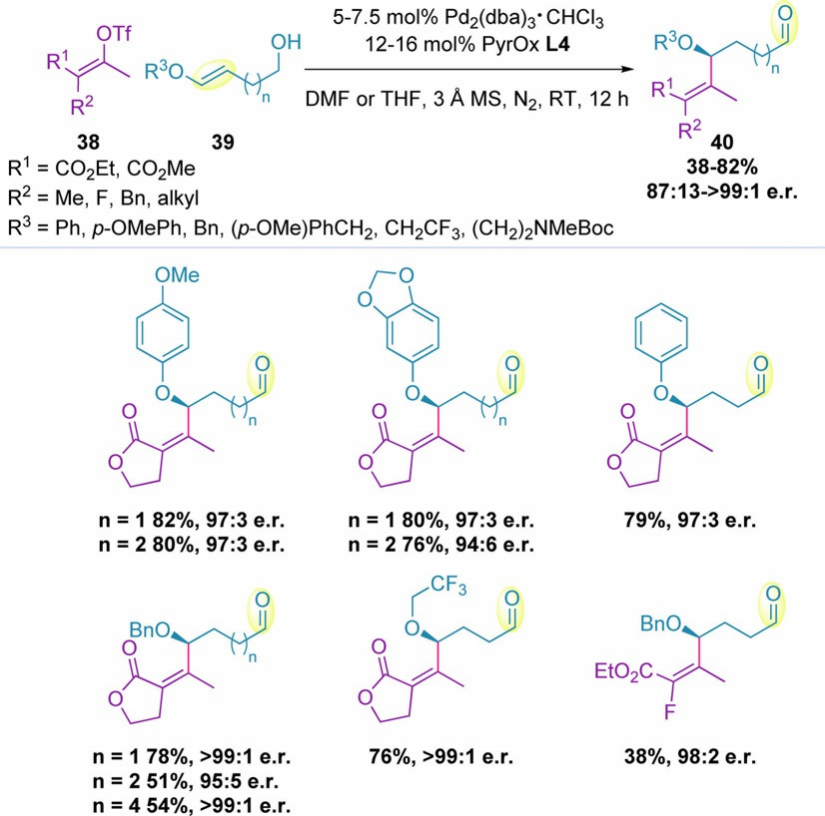
Alkenylation of acyclic aryl and alkyl enol ethers.

Acyclic aryl and alkyl enol ethers (**39**) can also react with alkenyl triflates (**38**) under similar conditions, forming chiral allylic ethers (**40**) (Scheme 11).^[41]^ Homoallylic‐ and longer chain‐alcohols could be utilized to generate the corresponding products in excellent enantiomeric ratios.

**Scheme 12:**
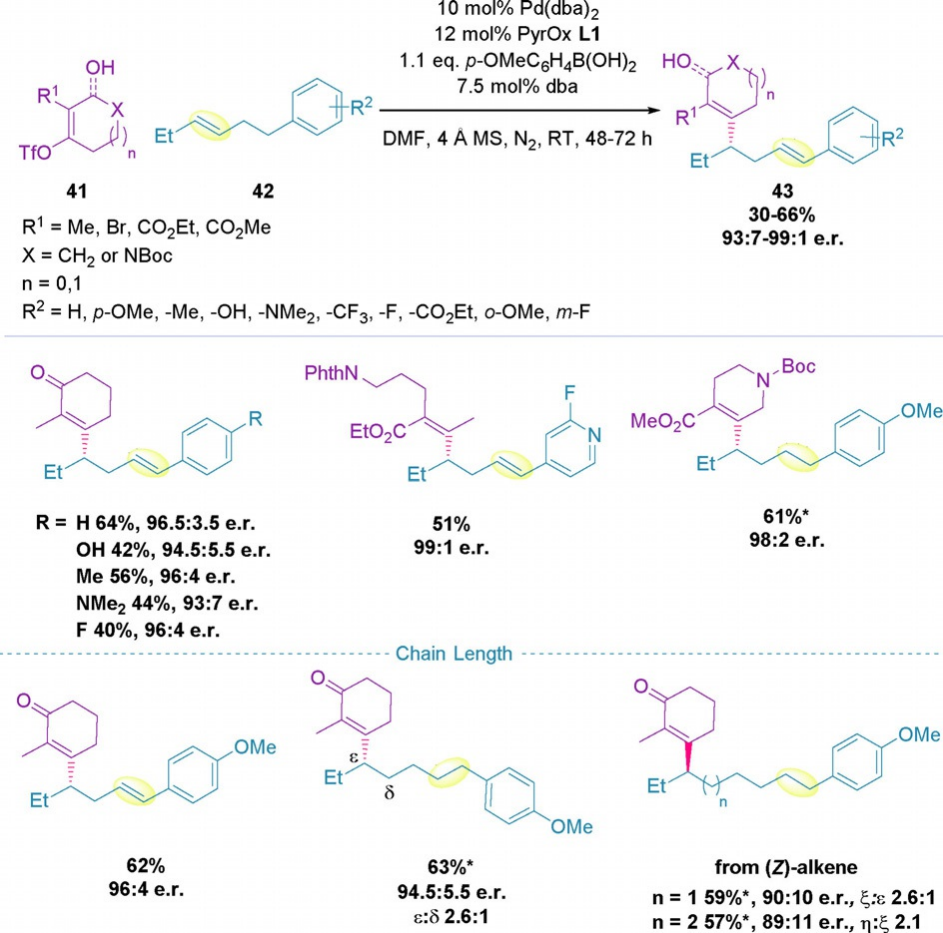
Redox‐relay Heck with alkenyl benzene derivatives.

It was later shown that alkenyl benzene derivatives (**42**) can successfully undergo chain walking events in the redox‐relay Heck coupling with alkenyl triflates (**41**).^[42]^ Here, the relay terminates to give the corresponding styrene products (**43**) in high enantiomeric ratios irrespective of the electronic nature of either coupling partner (Scheme 12). Whilst exploring the possibility of remote difunctionalization, it was noted that use of *para*‐methoxyphenyl boronic acid as an additive resulted in enhanced yields, although no difunctionalization was observed. Increasing the chain length led to a slight reduction in yield and enantioselectivity with each methylene addition. The site selectivities remained at 2.6:1 distal:proximal up to *tris*‐homoallylic alkenyl benzenes, but this diminished with addition of a further methylene unit.

### 2.4 Indoles

**Scheme 13:**
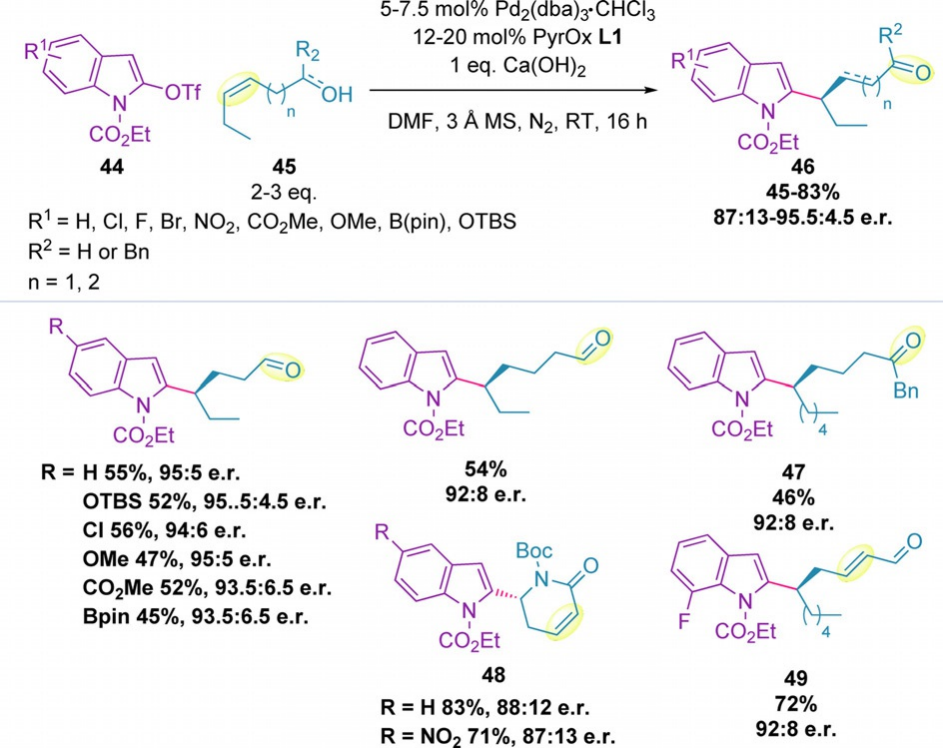
C2‐functionalization of indoles.

Indoles can be functionalized at the C2‐position via a redox‐relay Heck reaction by use of the corresponding triflate (Scheme 13).^[43]^ Only ethyl carbamate‐protected indoles (**44**) were successfully utilized, with methyl‐, phenyl‐ or acetyl‐protected indoles all resulting in either no product formation or decomposition. All substrates that coupled did so with high enantioselectivity, including one example with a secondary alcohol, which led to the formation of the corresponding ketone (**47**). These C2‐activated indole triflates could also be coupled to ene‐lactams (**48**) and disubstituted alkenes containing a remote carbonyl moiety, which results in formation of the corresponding α,β‐unsaturated product (**49**), in high enantiomeric ratios.^[43]^

### 2.5 Cyclic systems

**Scheme 14:**
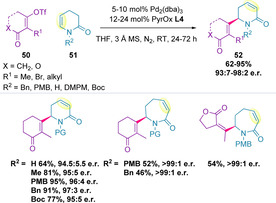
Alkenylation of ene‐lactams using electron‐deficient alkenyl triflates.

In 2019, the alkenylation of cyclic ene‐lactams (**51**) was reported (Scheme 14).^[44]^ Various *N*‐protecting groups were tolerated, generating the corresponding 6‐alkenyl substituted α,β‐unsaturated *δ*‐lactam **52** in excellent yields and enantioselectivities. Electron‐deficient alkenyl triflates could also be coupled successfully in high enantiomeric ratios. Attempts at expanding the scope to larger, seven‐membered ring systems saw enantioselectivity maintained on a reduced set of substrates, albeit with diminished yields, which could be recovered with increased catalyst loadings and longer reaction times.

## 3 The Redox‐Relay Oxidative Heck Reaction

**Scheme 15:**
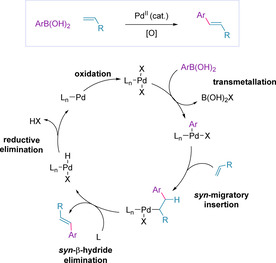
The oxidative Heck catalytic cycle.

The expansion of coupling partners beyond aryl diazonium salts led to the development of an effective complementary redox‐relay oxidative Heck variant.^[45]^ This oxidative Heck process uses a palladium(II) catalyst, and boronic acids replace the established unsaturated halides or triflates, thus making the first step of the catalytic cycle transmetallation as opposed to oxidative addition (Scheme 15).^[29]^ The term ‘oxidative‘ relates to the requirement for oxidation of Pd^0^ to Pd^II^ to regenerate the active catalytic species, and thus an oxidant is required in this case to achieve the desired transformation.[^29^, ^46^] This methodology has allowed more complex systems to be effectively cross‐coupled under milder conditions without the addition of base.[^46b^, ^47^]

### 3.1 Arylation

The seminal publication on the redox‐relay process by Sigman and co‐workers reported the addition of a broad scope of aryl boronic acids to a range of (*Z*)‐alkenols (**53**), forming the corresponding aldehyde (**54**/**55**) or ketone in high yield and enantioselectivity.^[45]^ When examining the impact of chain length, a similar trend to the redox‐relay Heck (see Section 2) was observed, where the site selectivity decreased with increased separation between the alkene and the alcohol, but enantioselectivity remained high (Table 4).

**Table 4:** Effect of chain length on site selectivity.

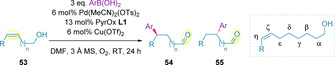					
Entry^[a]^	*n*	R	Yield [%]	e.r. (position)	**54/55**
1	0	Et	63	97:3 (β)	β:α>20:1
2	1	Me	50	98:2 (*γ*)	*γ*:β 6:1
3	2	Et	59	97:3 (*δ*)	δ:*γ* 7:1
4	3	Et	54	99:1 (*ϵ*); 99:1 (*δ*)	*ϵ*:*δ* 3.5:1
5	4	Et	63	97:3 (*ζ*); 99:1 (*ϵ*)	*ζ*:*ϵ* 3.2:1
6	5	Et	51	94:6 (*η*); 96:4 (*ζ*)	*η*:*ζ* 2.8:1

[a] Ar=(*p‐*CO_2_Me)Ph in all examples.

**Scheme 16:**
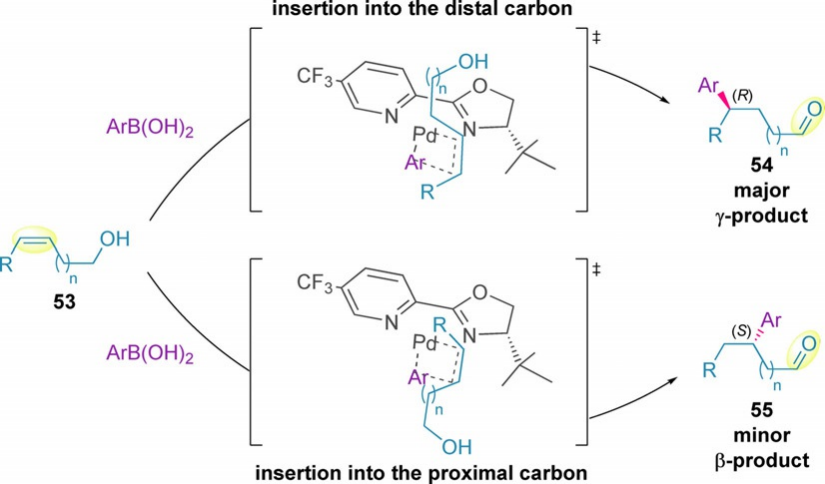
Addition of boronic acids to acyclic alkenols in the redox‐relay oxidative Heck.

Formation of the (*R*)‐stereocenter was favored at the distal alkene carbon. In contrast, for the minor product of the reaction, formed via insertion into the proximal carbon of the alkene, the (*S*)‐enantiomer was formed, implying that opposite faces of the alkene are presented to the palladium catalyst, as illustrated in Scheme 16.^[45]^ This rationale supports the high enantioselectivity observed experimentally for both products. It is worth noting that formation of the minor product also proceeds in high enantioselectivity despite migration occurring through the newly formed (*S*)‐stereocenter.

Replacing the alcohol with a carbonyl group (**56**) resulted in enhanced site selectivity for the corresponding α,β‐unsaturated products (**57**), compared to previous studies on systems of identical chain length (Table 5).^[48]^ A positive solvent effect was identified using DMA, resulting in *δ*:*γ* site selectivity as high as 15:1 (compared to 5.2:1 with DMF), whilst also increasing the yield and maintaining excellent enantioselectivity. The reaction performed well even in the absence of a co‐oxidant (meaning no additional solid oxidant was used), with comparable site selectivities to those reported previously. Elaboration of the carbonyl from a simple aldehyde (Entry 1) to ketones (Entry 2), carboxylic acids (Entry 3) and esters (Entries 4–6) was well tolerated. However, with increasing chain length, the site selectivity and yield were found to decrease (Entries 8–10).

**Table 5:** Effect of carbonyl functionalization and chain length on the redox*‐*relay oxidative Heck.

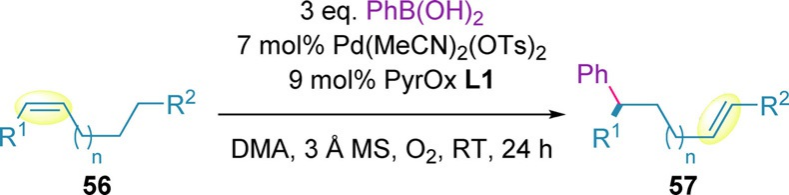						
Entry	*n*	R^1^	R^2^	Yield [%]	e.r.	Site selectivity
1	0	C_5_H_11_	CHO	78	99:1	*δ*:*γ* 15
2	0	C_5_H_11_	COMe	70	98:2	*δ*:*γ* 11
3	0	C_5_H_11_	COOH	61	97:3	*δ*:*γ* 7.9
4	0	C_5_H_11_	CO_2_Me	60	95:5	*δ*:*γ* 9.4
5	0	C_5_H_11_	CO_2_Et	61	96:4	*δ*:*γ* 8.5
6	0	C_5_H_11_	CO_2_^*i*^Pr	50	97:3	*δ*:*γ* 7
7^[a]^	0	C_5_H_11_	CN	47	95:5	*δ*:*γ* 16
8	0	Et	CHO	50	98:2	*δ*:*γ* 6.1
9	2	Et	CHO	41	93:7	*ζ*:*ϵ* 2.1
10	3	Et	CHO	37	98:2	*η*:*ζ* 1.5

[a] *E*:*Z=*5:1. All other entries showed an *E*:*Z*>20:1.

**Scheme 17:**
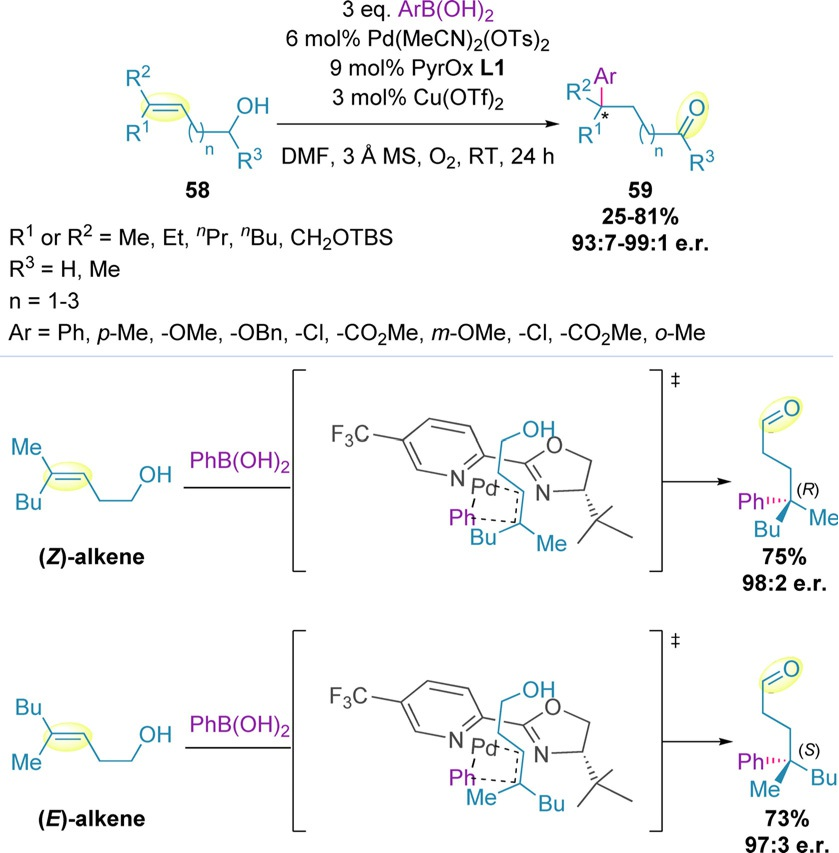
Impact of alkene geometry of trisubstituted alkenols on enantioselectivity.

The scope was later expanded to trisubstituted alkenols (**58**), forming the corresponding aldehydes (**59**), containing a remote quaternary stereocenter, in high enantiomeric ratio (Scheme 17).^[49]^ Unlike the majority of disubstituted alkenols, trisubstituted alkenols showed high site selectivity (γ:β≥15:1) regardless of the electronic nature of the boronic acid. This enhanced selectivity is attributed to the more nucleophilic nature of the alkenol. For these systems, high site‐ and enantio‐selectivity was also achieved irrespective of the chain length. The degree of enantioselectivity was found to be the same regardless of alkene geometry, but with the formation of opposite enantiomers as the major product. A rationale for this was proposed based on the transition state for migratory insertion, in which the binding orientation of the alkene is unchanged for both alkene configurations (Scheme 17).[^49^, ^50^]

**Scheme 18:**
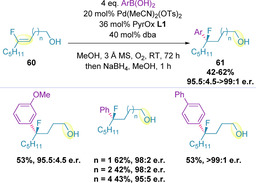
Arylation of fluorine‐containing alkenols.

Arylation of trisubstituted (*Z*)‐alkenols (**60**) containing a fluorine substituent on the alkene has also been demonstrated, although the majority of the investigation focused on β‐functionalization.^[51]^ Substantially higher catalyst loadings were required to generate more remote fluorinated tertiary stereogenic carbon centers (**61**), but these were found to proceed in excellent enantioselectivity, which diminished slightly with increased chain length (Scheme 18). This methodology enables the straightforward access to enantio‐enriched fluorinated building blocks, which, in some cases, are known to confer favorable physicochemical properties in drug molecules.^[52]^

**Scheme 19:**
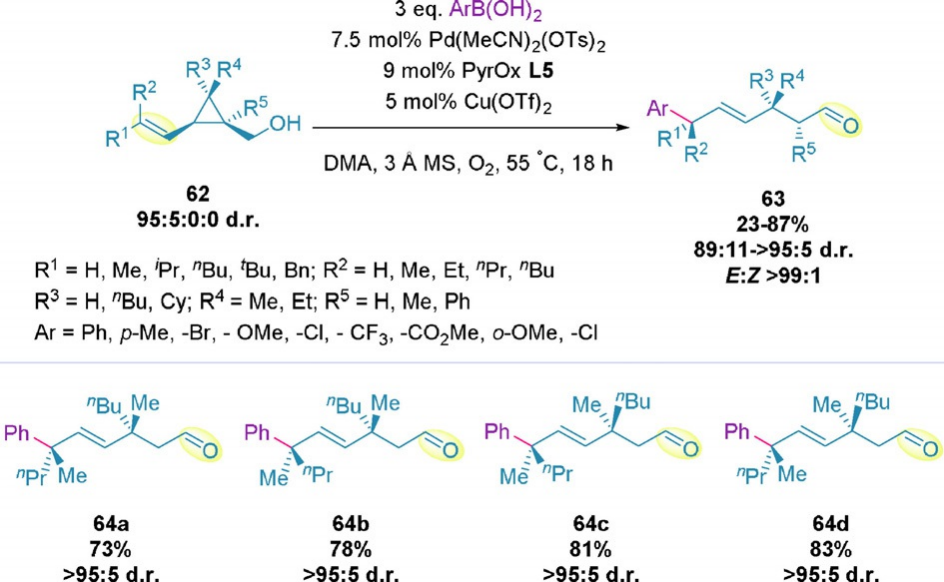
Generation of multiple tertiary and quaternary stereocenters by redox‐relay oxidative Heck cyclopropane ring opening.

The presence of quaternary stereocenters along the acyclic chain is incompatible with the intrinsic mechanism of chain walking events. Inspired by their earlier work, Marek and co‐workers exploited the possibility of halting the migration by installing a cyclopropane ring bearing a quaternary stereocenter along the chain.^[53]^ This enabled a ring‐opening event during migration, generating acyclic systems bearing multiple congested stereogenic elements (**63**) in four steps, with a high degree of stereocontrol (Scheme 19).^[54]^ Although the chiral vinylcyclopropyl carbinol precursor (**62**) governed the stereochemical outcome, PyrOx **L5** (Figure 1) was found to promote a more selective migratory insertion, which led to increased yields.

Di‐ and tri‐substituted alkenes were compatible with this process, producing tertiary and quaternary stereocenters, respectively. However, the authors found that bulkier chains on the alkene led to slightly diminished diastereo‐ and regio‐selectivities. 1,1,2,2,3‐Pentasubstituted vinylcyclopropyl carbinols (**62**, where R^5^≠H) were also amenable to this protocol, generating products with three stereogenic centers, without epimerization at the α‐stereocenter. In terms of boronic acid scope, a variety of functional groups at the *para*‐ and *meta*‐position were tolerated, including ethers, halides, esters, ketones and nitro groups, whereas, *ortho*‐substituted substrates proved inefficient, and heteroaryl boronic acids proceeded in lower yield. This methodology is also compatible with triflates and indoles as coupling partners. The reaction is stereospecific, meaning that diastereomeric products can be accessed by altering the alkene geometry. This powerful feature of the process was fully exploited in the synthesis of all four diastereomers (**64 a** to **64 d**) of precursor **62** (Scheme 19). Identical levels of selectivity were observed for both alkene geometries.

### 3.2 Indoles

**Scheme 20:**
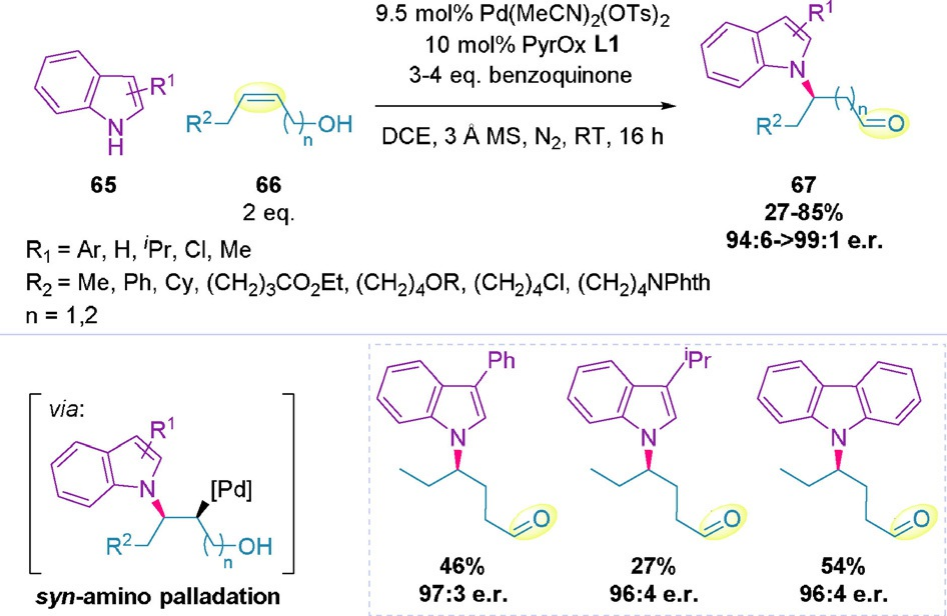
*N*‐alkylation of indoles.

The enantioselective *N*‐alkylation of indoles (**65**) to (*Z*)‐alkenols (**66**) has recently been reported by Sigman and co‐workers (Scheme 20).^[55]^ This transformation can be formally classified as an intermolecular aza‐Wacker reaction, whereby selective β‐hydride elimination prevents the formation of the corresponding enamine and instead leads to aldehyde **67** via chain walking migration. Mechanistic experiments carried out by the authors support a *syn*‐amino‐palladation pathway. The majority of substrates generated using this methodology demonstrate β‐functionalization. Although high stereoselectivities are consistently achieved, an excess of **66** is required in order to ensure reaction conversion.

**Scheme 21:**
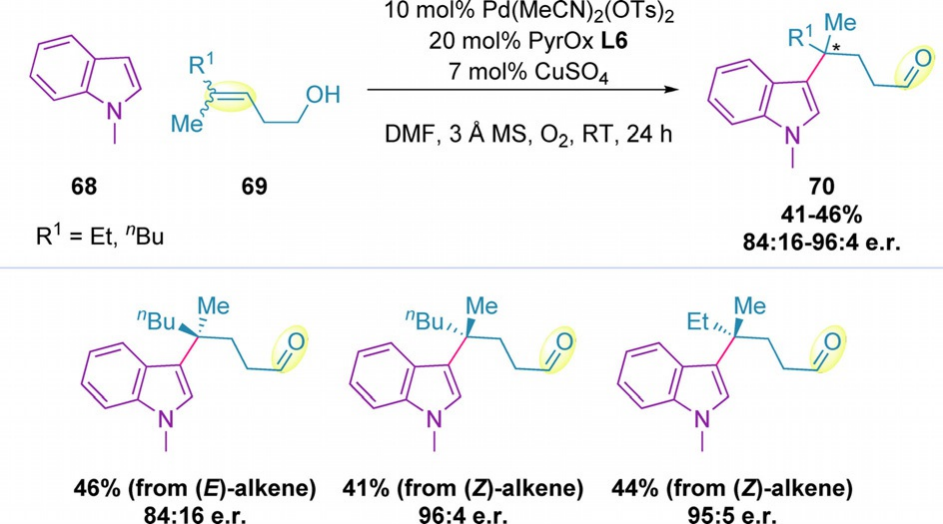
C3‐functionalization of indoles.

A complementary C3‐functionalization of indole **68** via a dehydrogenative redox‐relay oxidative Heck strategy to generate quaternary stereocenters (**70**) has also been developed (Scheme 21).^[34]^ Again, the majority of the scope focused on β‐functionalization, but selected examples demonstrate more remote functionalization. Whilst both alkene isomers resulted in comparable yields, the enantioselectivities were significantly greater for (*Z*)‐alkenes.

### 3.3 Cyclic systems

**Scheme 22:**
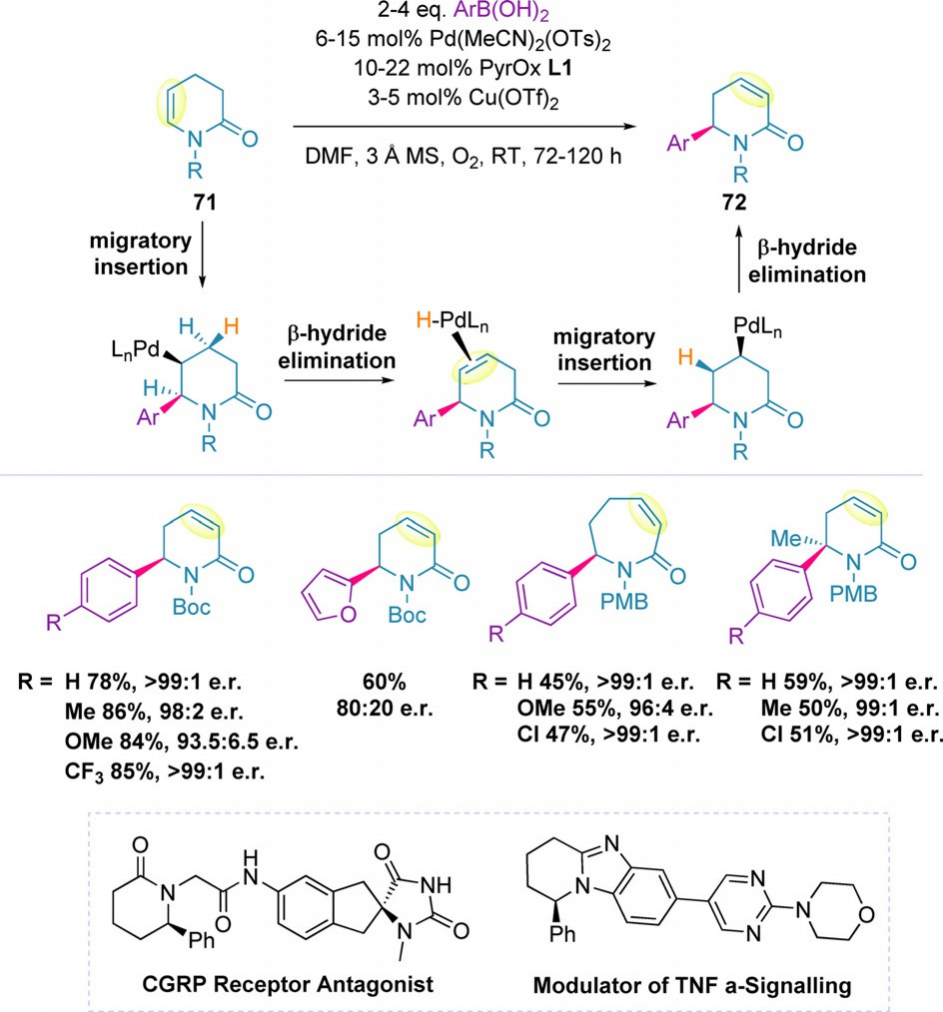
Redox‐relay oxidative Heck reaction *Scheme*, mechanism and scope for the lactam system.

In 2018, Sigman and co‐workers began to investigate the redox‐relay oxidative Heck reaction with cyclic systems, focusing on lactams (**71**).^[56]^ Regioselective insertion using PyrOx **L1** led to the monoarylated ene‐lactam product (**72**) (Scheme 22). This procedure was compatible with a variety of nitrogen protecting groups (Boc, Bn, Ts, Me and PMB) as well as the unprotected lactam, with high enantioselectivities (95:5‐>99:1 e.r.*)* achieved in all cases. Application of a range of electron‐rich and electron‐deficient aromatics proceeded to give the corresponding *δ*‐lactam product in high yield and enantiomeric ratios, although heteroaromatic and electron‐rich boronic acids generally correlated with lower enantioselectivities.

Expanding the ring size to produce *ϵ*‐lactams required higher catalyst loadings due to the diminished activity of these substrates. This produced the desired products in high enantiomeric ratio but lower yield. The scope of this methodology also encompassed the formation of quaternary stereogenic centers from trisubstituted ene‐lactams bearing a methyl substituent. Excellent enantiomeric ratios were demonstrated in all cases, regardless of the electronic nature of the boronic acid substrate. More sterically demanding trisubstituted ene‐lactams did not yield any of the desired product. Chiral building blocks for a variety of drug molecules, such as a CGRP receptor antagonist and a modulator of TNF α‐signalling, have been generated from the arylation of ene‐lactams in high yield and with no erosion of enantioselectivity over subsequent steps.^[56]^

**Scheme 23:**
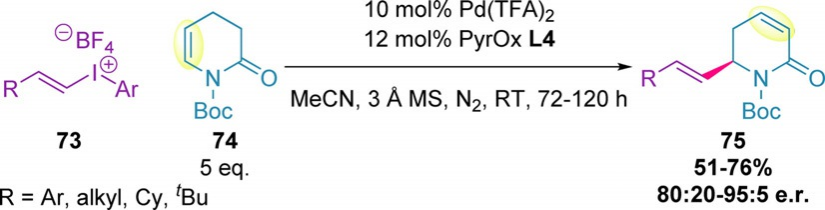
Alkenylation of ene‐lactams using electron‐rich iodonium salts.

In a complementary study, tetrafluoroborate iodonium salts (**73**) were found to be most efficacious for the alkenylation of ene‐lactams (**74**), allowing electron‐rich alkenyl groups to be used (Scheme 23).^[44]^ Given the challenging nature of this transformation the yields and enantioselectivities achieved were remarkable.

**Scheme 24:**
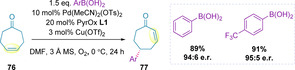
Arylation of cycloheptenones.

Investigation into the redox‐relay oxidative Heck methodology has since progressed onto less biased systems, such as symmetrical cycloheptenones (**76**) (Scheme 24).^[57]^ In this case, remote arylation proceeded in high yield and enantioselectivity. Of note, longer reaction times or greater equivalents of boronic acid led to increased levels of the diarylated product. Alkenylation was also possible with alkenyl triflates, using a standard redox‐relay Heck protocol, albeit in lower yields and more modest enantiomeric ratios. Attempts to explore unsymmetrical cycloalkenones resulted in poor site selectivity.

## 4 Mechanistic Investigations

**Scheme 25:**
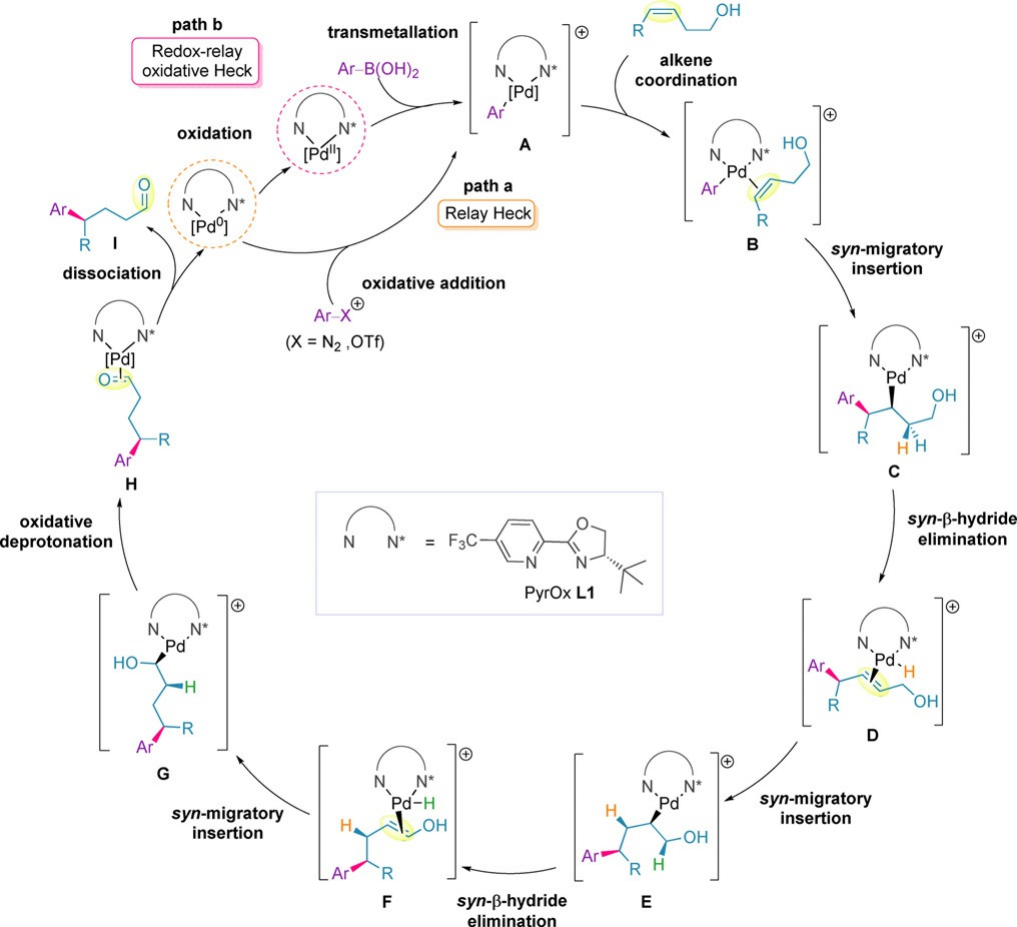
Proposed catalytic cycle for the redox‐relay Heck (path a) and oxidative Heck (path b) reactions based on empirical and computational studies.

Since their discovery, considerable efforts to develop a fundamental understanding of both redox‐relay Heck and oxidative Heck transformations have been undertaken.[^50^, ^58^] The reaction is thought to proceed via migratory insertion of the aryl (or vinyl) coupling partner onto the distal carbon of the alkene, relative to the alcohol. Successive β‐hydride elimination/migratory insertion steps then lead to the product. Mechanistic studies, reinforced by computational calculations, have been carried out to support a proposed catalytic cycle for alkenol substrates (Scheme 25).[^50^, ^58^]

### 4.1 Oxidative addition or transmetallation

**Scheme 26:**
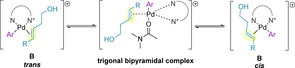
Berry pseudorotation of species **B**.

Initially, the palladium(0) catalyst undergoes ligand substitution with the chiral PyrOx ligand.^[58a]^ The catalytic cycle then begins with either the oxidative addition of the aryl diazonium salt to the palladium(0) catalyst in the Heck reaction, or transmetallation of the aryl boronic acid to the palladium(II) catalyst in the oxidative Heck reaction, forming palladium(II) species **A**. A π‐complex with the alkene of the alkenol is then formed (species **B**).[^50^, ^58a^] Computational studies have found that both the oxidative addition of aryl diazonium salts in the Heck reaction; the transmetallation of aryl boronic acids in the oxidative Heck; and the subsequent alkene coordination steps, are all energetically facile.^[59]^ Kinetic studies for the oxidative Heck indicate a fast transmetallation of the boronic acid prior to rapid alkene binding.^[60]^ A feasible low energy associative mechanism for isomerization of **B** in the presence of DMF was found, indicating that both *cis*‐ and *trans*‐isomers of **B** exist in rapid equilibrium via a trigonal bipyramidal intermediate (Scheme 26).^[50]^

### 4.2 Migratory insertion

It is proposed that with rapid isomerization of **B**, the subsequent *syn*‐migratory insertion of the alkenol should be under Curtin–Hammett control, determined by the relative energy of insertion transition states that exist for both isomers. The large (10.7 kcal mol^−1^) energy barrier for migratory insertion suggests that this step in the mechanism is responsible for determining the site‐ and enantio‐selectivity of the product.^[50]^ It was found that the redox‐relay pathway is of lower energy, therefore favoring this process over the traditional Heck manifold.[^50^, ^58a^] It was also noted that the traditional Heck pathway is reversible.

### 4.3 Redox‐relay chain walking

Experimental observations on the systems studied are suggestive of palladium chain walking events.[^49^, ^56^] Similar observations have previously been made with systems involving the cycloisomerization of 1,*n*‐dienes.^[20]^ This is also in agreement with the computational studies, which indicate that, following the formation of **C**, site‐selective β‐hydride elimination leads to intermediate **D**.[^50^, ^58a^] The large free energy of activation calculated suggests that this step is turnover‐limiting.^[58a]^ This β‐hydride elimination is accompanied by solvent mediated *cis‐trans* isomerization in the lower energy pathway to relieve strain. Calculations to determine if displacement of the cationic palladium from the alkene occurs, resulted in an endothermic pathway, suggesting that this process is unlikely. The authors postulate that this is due to the electrophilic nature of the chiral ligand, reinforcing that this property is key to the development of an efficient transformation. In addition, the lack of a strong base in this methodology prevents deprotonation, thus allowing reinsertion to occur, forming intermediate **E**. A further β‐hydride elimination results in the formation of enol **F**.

**Scheme 27:**
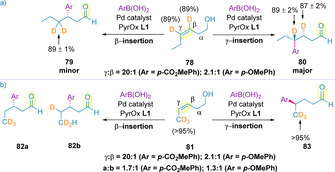
Deuterium labelling experiments as evidence for the uni‐directional nature of chain walking events.

The aforementioned chain walking events (**C** to **F**) have low calculated energy barriers, suggesting that such steps may be reversible.[^49^, ^50^, ^58^] Deuterium‐labelling experiments were also carried out to further probe this aspect of the mechanism. Retention of *γ*‐ and β‐deuterium labels in **78** was observed experimentally, implying preferential migration of the catalyst towards the alcohol in formation of the major product (**80**) (Scheme 27 a). This observation is in agreement with the computationally‐determined mechanism.^[58b]^ For the minor product of the reaction (**79**), only deuterium incorporation at the distal carbon of the alkene was observed, again suggesting that the relay is uni‐directional. Deuterium labelling of the terminal methyl of the alkenol (**81**) also resulted in the same conclusion for *γ*‐arylation (**83**) (Scheme 27 b). However, for β‐arylation, deuterium scrambling was observed (**82 a** and **82 b**), indicating that migration through a sterically‐hindered benzylic position enables reversible chain walking that still ultimately leads to migration towards the alcohol redox acceptor, forming the observed carbonyl product.^[58b]^

**Scheme 28:**
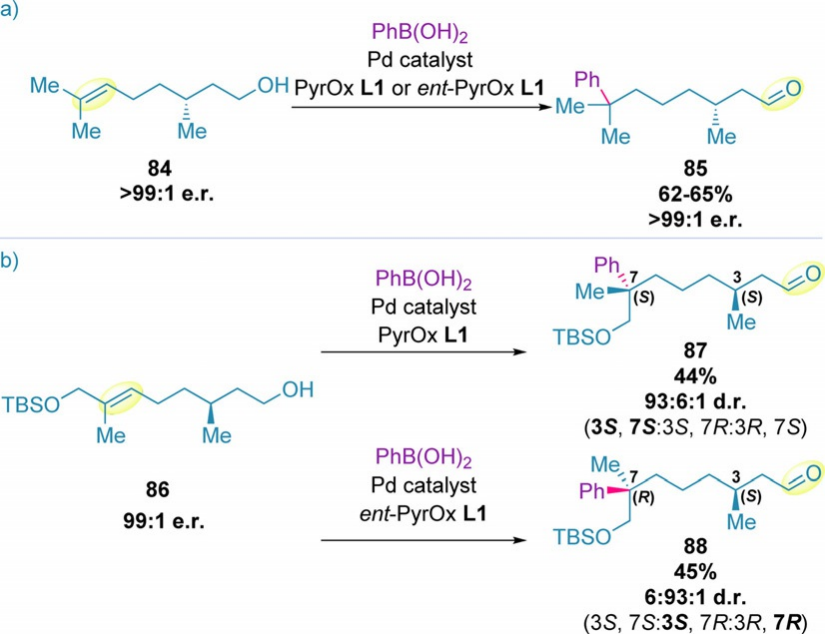
Experiments with pre‐installed stereocenters.

The reaction of trisubstituted alkenol **84**, which contains a pre‐installed stereocenter on the alkyl chain, in the presence of either enantiomer of the chiral PyrOx ligand (**L1** or ***ent*** **‐L1**), resulted in formation of the same product (**85**) in high enantioselectivity and identical yield (Scheme 28 a).^[49]^ The integrity of the pre‐installed stereocenter was preserved, implying that the catalyst remains associated on the same face of the alkenol substrate during the relay process. Repeating these control experiments with alkenol **86** resulted in high diastereoselectivity, but giving different diastereomers as the major product (**87** and **88**) (Scheme 28b). This observation supports a catalyst‐controlled face selection of the alkene. Further deuterium labelling studies identified that the palladium species remains associated with the alkenol during migration, thereby providing additional evidence that reinsertion of the palladium occurs on the same face of the alkene.^[58a]^

### 4.4 Dissociation of product

**Scheme 29:**
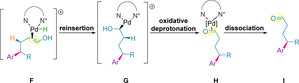
Mechanism for product dissociation proposed by Sigman and co‐workers.

Several theories have been proposed to rationalize the final dissociation step of the mechanism.[^50^, ^58^] Sigman and co‐workers suggested that reinsertion of the palladium hydride forms hydroxyalkyl‐palladium species **G**, which acts as a thermodynamic sink in the chain walking mechanism (Scheme 29).^[61]^ The authors state that the presence of the palladium species **G** rules out a possible tautomerization mechanism of the enol to the carbonyl proposed by Wang and co‐workers.^[58b]^ It has been found that with successive migratory insertion/β‐hydride elimination steps, as the palladium species migrates closer towards the oxygen of the alcohol, the energy barrier decreases. This shallow potential energy surface represents an energetically downhill process, and is proposed to be due to a favorable interaction between the partial negative charge of the palladium‐bound carbon and the partial positive charge of the oxygen‐bound carbon.[^50^, ^58b^] Formation of **G** is then followed by an oxidative deprotonation of the alcohol by DMF, which leads to formation of the carbonyl product bound to the regenerated Pd^0^, species **H**.^[50]^ Dissociation of the catalyst releases the product **I**, completing the catalytic cycle for the redox‐relay Heck. For the redox‐relay oxidative Heck, the palladium(0) species is then re‐oxidized to Pd^II^ by an oxidant to complete the catalytic cycle.

### 4.5 Further mechanistic exploration

**Scheme 30:**
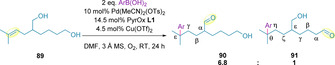
Internal relay competition showing preferential migration of the catalyst to the nearest alcohol.

Performing the redox‐relay methodology on diol **89** enabled further study of the relay mechanism.^[60]^ Specifically, the presence of two alcohols of varying distance from the alkene, stemming from a branch point in substrate **89**, would allow any electronic influence of each alcohol on the migration to be probed. It was found that the catalyst migrates preferentially towards the closest alcohol, resulting in the formation of aldehyde **90** over **91** in a ratio of 6.8:1 (Scheme 30). This observation was supported by computational studies, where a progressively lower energy barrier was observed as the palladium center migrates towards the alcohol.[^50^, ^58^, ^60^]

### 4.6 Enantioselectivity

**Figure 2:**
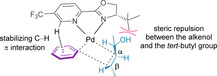
Transition state for migratory insertion with PyrOx **L1**.

Computational studies suggest a turnover‐determining arylation step, followed by successive β‐hydride elimination and migratory insertion steps before release of the product.^[58a]^ As discussed in Section 4.2, it is the initial migratory insertion of the arene which was determined to be both the enantio‐ and regio‐determining step.[^50^, ^58a^, ^60^] The broad substrate scope demonstrated in the literature for these reactions supports the notion that the electronic and steric attributes of both the alkenol and arene coupling partners have little effect on enantioselectivity.^[49]^ The enantioselectivity is instead believed to be controlled by steric repulsion between the alkenol and the *tert*‐butyl group on the chiral PyrOx ligand (**L1**), as well as a stabilizing C−H π interaction of the pyridinyl ring of the chiral ligand and the arene (Figure 2).[^50^, ^58a^] The *trans* influence is also assumed to operate, with the natural bond orbital charge of the oxazoline nitrogen thought to make it a stronger σ‐donor than the nitrogen of the pyridine.^[58a]^ The computationally calculated enantioselectivities (>99 %) are in good agreement with experimentally determined values.

In further studies on alkenol systems, it has also been found that polarization of the alkyl chain, as a result of the distance between the alkene and the alcohol, aids in face discrimination of the boronic acid substrate during the rate‐determining migratory insertion step.^[62]^ As enantioselectivity was found to decrease with increasing chain length for this system, it was proposed that a lone pair‐π interaction between the alcohol and the aryl group on the oxazoline ring is predominantly responsible for the high levels of enantioselectivity. This favorable interaction is weakened with increasing chain length, leading to lower enantioselectivities.

### 4.7 Site selectivity

Whilst the electronic and steric properties of the coupling partners do not have a strong influence on enantioselectivity, they were found to govern the site selectivity. For alkene insertion, the aryl group can be either *cis* or *trans* in relation to the oxazoline group of the chiral ligand.^[50]^ For these configurations, the aryl group can insert at either the *γ*‐(distal) carbon or β‐(proximal)carbon from either face of the alkene, leading to (*R*)‐ or (*S*)‐stereochemistry in the product.

**Figure 3:**
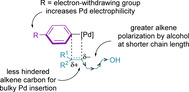
Factors governing site selectivity for the redox‐relay (oxidative) Heck.

It was determined that the site selectivity is controlled by the difference in electronic structure between the two sp^2^‐carbons of the alkene.[^49^, ^50^] Interaction with the electrophilic palladium leads to polarization of the alkene in the transition state (Figure 3).[^50^, ^58a^] This results in the carbon atom of the nascent Pd−C bond being more negatively charged, whilst there is a simultaneous build‐up of positive charge on the adjacent carbon atom where the aryl group will subsequently insert.[^45^, ^50^, ^58a^, ^63^] This site selectivity also aids in minimizing steric repulsion, as the bulky palladium catalyst is situated on the less hindered alkene carbon. This theory is in agreement with experimental evidence that site selectivity increases with decreasing electron density of the arene, increased steric demand around the alkene, and a shorter chain length. Other factors, such as the reaction solvent, have also been found to influence the observed site selectivity.^[48]^

## 5 Applications in Synthesis

While reports on redox‐relay (oxidative) Heck reactions have frequently applied these methods to pharmaceutically relevant structures to highlight their potential utility, there have been fewer examples of widespread application beyond those used to showcase the specific developed methodology.

**Scheme 31:**
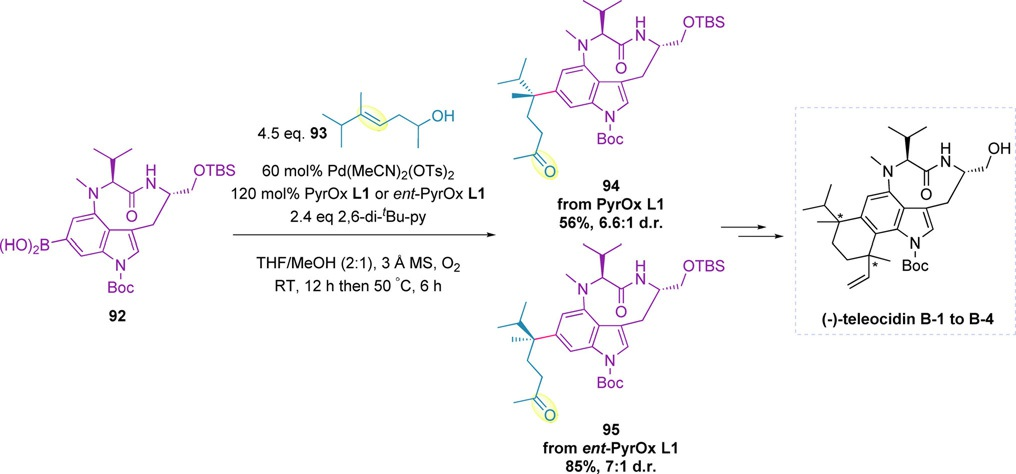
Synthesis of (−)‐teleocidin B‐1 to B‐4 using a redox‐relay oxidative Heck diversification strategy.

Baran and co‐workers have employed the redox‐relay oxidative Heck in the 11‐step synthesis of natural products (−)‐teleocidin B‐1 to B‐4 (Scheme 31).^[64]^ The coupling of intermediate boronic acid **92** with (*E*)‐alkenol **93** resulted in construction of a key quaternary stereocenter in the synthesis. By using both enantiomers of PyrOx ligand (**L1** and ***ent*** **‐L1**) in turn, it was possible to synthesize both diastereomers of the product (**94** and **95**). Subsequent formation of the second quaternary stereocenter meant that all four diastereomers (B‐1 to B‐4) could be synthesized from the same starting materials. Compared to prior reports,^[49]^ significantly higher catalyst and ligand loadings were required, as well as an increased excess of the alkenol to be coupled. Addition of 2,6‐di‐*tert*‐butylpyridine (2,6‐di‐*t*Bu‐py) was also required, to reduce an undesired protodeborylation side reaction.

**Scheme 32:**
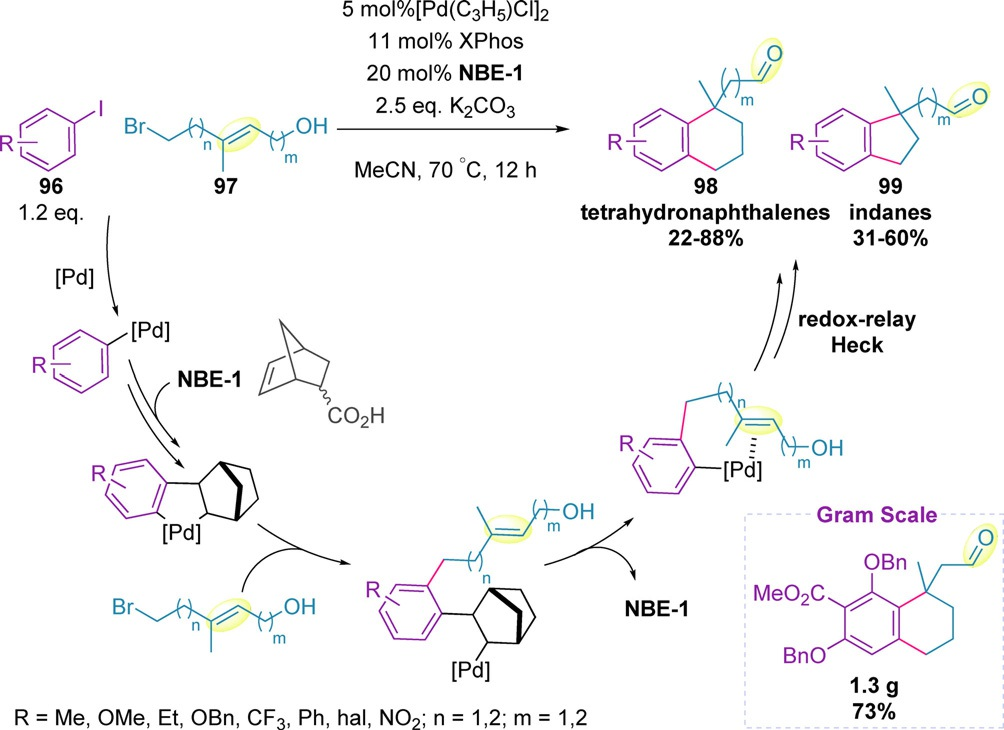
Catellani redox‐relay Heck reaction.

The Catellani reaction is a palladium‐catalyzed *ortho* C−H functionalization of an aryl halide mediated by norbornene, followed by cross‐coupling at the *ipso*‐position.^[65]^ A tandem Catellani redox‐relay Heck reaction has been developed by Zhou and co‐workers for the synthesis of pharmaceutically relevant tetrahydronaphthalene (**98**) and indane (**99**) scaffolds, utilizing a norbornene (**NBE‐1**) derived mediator (Scheme 32).^[66]^ During optimization a notable difference in reactivity between (*E*)‐ and (*Z*)‐alkenols was identified (81 % vs. 67 % respectively) and so only (*E*)‐alkenols (**97**) were examined in the substrate scope. A variety of electron‐donating and electron‐withdrawing aromatics were compatible with this methodology. Furthermore, heterocycles (pyridine and isoquinoline) were also in scope. The synthetic utility of the transformation was further demonstrated by performing a reaction on gram‐scale in comparable yield.

**Scheme 33:**
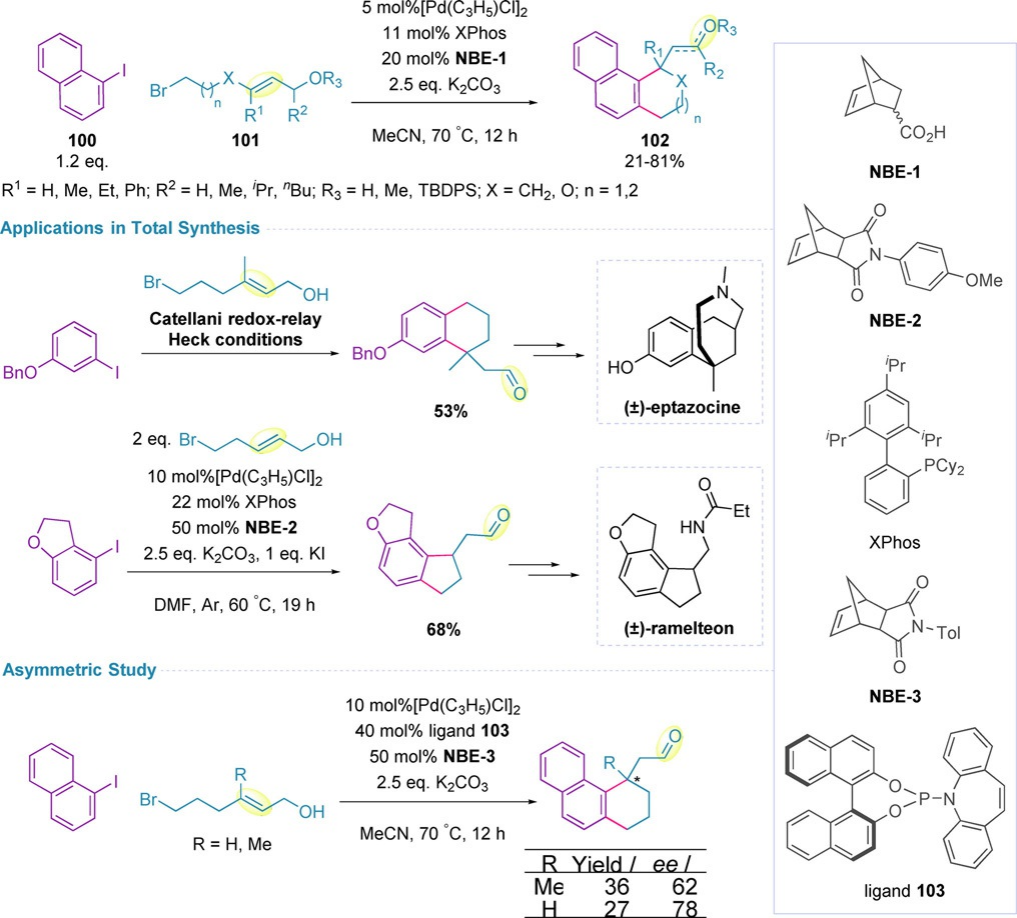
Application of the Catellani redox‐relay Heck reaction in natural product and drug synthesis.

It was also reported that secondary allylic alcohols (**101**, where R^3^=H) were successful in generating the corresponding ketone (Scheme 33, 45–71 %), whilst protected alcohols (**101**, where R^3^≠H) afforded the corresponding enol ethers in good yield (60–81 %). This strategy has subsequently been applied in the four‐step total synthesis of (±)‐eptazocine (previously ≥7 steps), showcasing that more complex quaternary benzylic stereocenters, prevalent in a number of bioactive molecules, can be generated efficiently. Later, a modified set of conditions were employed in the three‐step total synthesis of (±)‐ramelteon (previously ≥4 steps).^[67]^ Attempts to establish an asymmetric variant of this methodology have been initiated, so far with only limited success and moderate enantiocontrol (Scheme 33).

## 6 Summary and Outlook

Since the seminal publication first reporting the unexpected chain walking reaction in 1968, the field of palladium catalyzed redox‐relay Heck migration has evolved significantly. Over the past decade the comprehensive development of asymmetric redox‐relay Heck reactions and its oxidative variant using PyrOx ligands has led to an ever‐increasing understanding of their synthetic potential. Such transformations typically exhibit high levels of regio‐ and enantio‐control, thus providing easy access to novel chiral building blocks bearing remote tertiary or quaternary stereocenters that would otherwise be challenging to synthesize.

Whilst substantial advances are being made in the field to make this methodology more widely applicable, extensive work will undoubtedly be required to understand the subtleties of the migration on more complex and non‐biased systems. In time, these discoveries should aid in making new, complex transformations more predictable and reliable. This is a major research focus, and efforts towards predicting enantioselectivities have already been reported.^[68]^ The potential scalability of this transformation has also yet to be thoroughly investigated, and detailed studies from this perspective may encourage industrial uptake of the methodology.^[69]^ For the redox‐relay Heck reaction, additional considerations need to be taken for the use of diazonium salts in palladium catalysis,^[70]^ whilst there is added complexity in the redox‐relay oxidative Heck reaction based on the greater number of additives, which could make it more challenging to understand the role of all reaction components. In addition, the use of oxygen is undesirable on scale.^[71]^ Another avenue which may provide new synthetic opportunities include one‐pot sequential catalysis, whereby the in situ generated redox‐relay product could be subsequently cross‐coupled.^[72]^

From a synthetic utility viewpoint, the expansion of scope to a wider range of heterocycles, as well as additional redox acceptors capable of driving the chain migration such as sulfones and sulfonamides, would make this transformation more broadly applicable for the synthesis of bioactive small molecule libraries in drug discovery.^[73]^ Another limitation is the synthesis of quaternary stereocenters, which is currently restrained by the steric hindrance of the alkene substituents.[^8d^, ^56^] It is worth noting that there have been multiple reports on redox‐relay (oxidative) Heck β‐functionalization using carbamates as coupling partners;^[74]^ protected alcohols to furnish the corresponding carbonyl products, via in situ deprotection strategies;[^61^, ^75^] or alkynes to generate C_sp_–C_sp_^3^ stereocenters.^[76]^ Expansion of these methodologies for more remote functionalization would also be of great value. It is our hope that the synthetic utility of the transformations and applications described in this Minireview will inspire future research in this field and in turn help address the current limitations discussed.

## Conflict of interest

The authors declare no conflict of interest.

## Biographical Information

*Holly Bonfield received her MChem degree from the University of York*, *UK. In 2018*, *she joined the University of Strathclyde/GlaxoSmithKline industrial PhD programme under the joint supervision of Drs David Lindsay*, *Marc Reid and Damien Valette. Her research focuses on synthetic applications of asymmetric remote functionalization methods for the synthesis of bioactive molecules*.

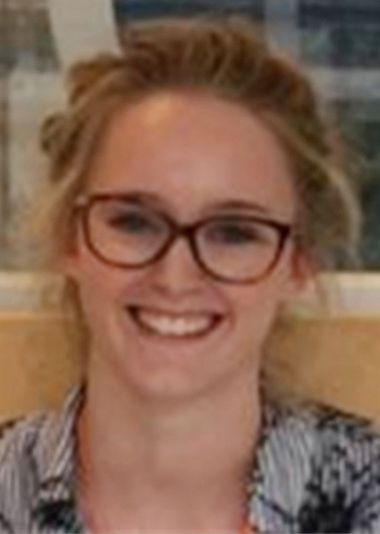

## Biographical Information

*Damien Valette obtained his PhD from the University of Sheffield*, *UK in 2011 under the supervision of Prof. Simon Jones. He subsequently joined the laboratory of Prof. Huw M. L. Davies at Emory University*, *USA as a postdoctoral fellow where he studied catalytic asymmetric C−H functionalization transformations. In 2013 he started his industrial career in the second‐generation process development department at Pfizer (Cork*, *Ireland). Since 2014 he works on the development of new synthetic routes of APIs in both chemical development and medicinal chemistry departments at GlaxoSmithKline (Stevenage*, *UK)*.

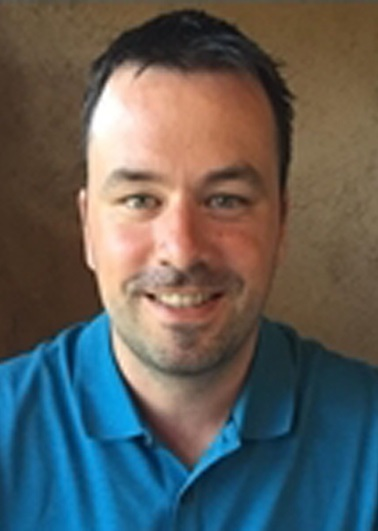

## Biographical Information

*David Lindsay received his PhD from the University of Strathclyde in 2000*, *under the supervision of Prof. William J. Kerr. Following postdoctoral research*, *first as an Alexander von Humboldt Fellow with Prof. Paul Knochel at Ludwig‐Maximilans‐Universität in Munich*, *and then with Prof. Tim Gallagher at the University of Bristol*, *he took up an EPSRC Advanced Research Fellowship at the University of Bristol. After posts at the Universities of Reading and Glasgow*, *he moved to his present position at the University of Strathclyde in 2014. His research interests lie in the application of organometallic complexes in synthesis*, *catalysis*, *and materials chemistry*.

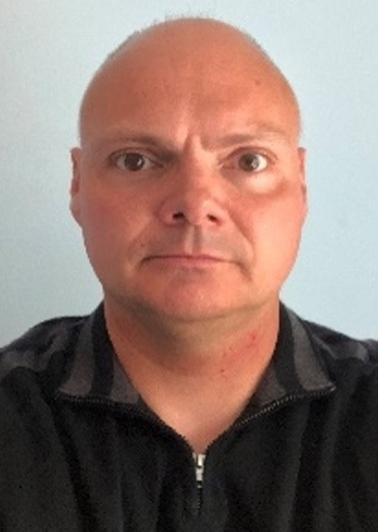

## Biographical Information

*Marc obtained his PhD at the University of Strathclyde in 2015*, *under the joint supervision of Professors William J. Kerr and Tell Tuttle. He completed a postdoctoral appointment with Professor Guy Lloyd‐Jones at the University of Edinburgh before returning to Strathclyde to start his independent career with a GSK‐supported Leverhulme Trust Early Career Fellowship. In 2020*, *he became a Lecturer at the University of Bristol. Outside academia*, *Marc is the founder of Pre‐Site Safety. His research interests span physical organic chemistry*, *computer vision*, *virtual reality*, *and process safety*.

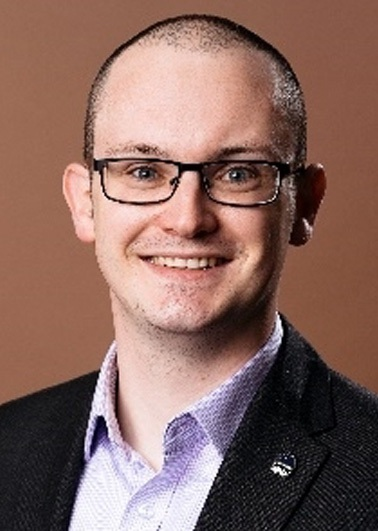

## References

[1] D. G. Brown , J. Bostrom , J. Med. Chem. 2016, 59, 4443–4458. 26571338 10.1021/acs.jmedchem.5b01409

[2a] Y. Liu , S. J. Han , W. B. Liu , B. M. Stoltz , Acc. Chem. Res. 2015, 48, 740–751; 25715056 10.1021/ar5004658 PMC6410712

[2b] S. M. Mennen , C. Alhambra , C. L. Allen , M. Barberis , S. Berritt , T. A. Brandt , A. D. Campbell , J. Castañón , A. H. Cherney , M. Christensen , D. B. Damon , J. Eugenio de Diego , S. García-Cerrada , P. García-Losada , R. Haro , J. Janey , D. C. Leitch , L. Li , F. Liu , P. C. Lobben , D. W. C. MacMillan , J. Magano , E. McInturff , S. Monfette , R. J. Post , D. Schultz , B. J. Sitter , J. M. Stevens , I. I. Strambeanu , J. Twilton , K. Wang , M. A. Zajac , Org. Process Res. Dev. 2019, 23, 1213–1242;

[2c] K. R. Campos , P. J. Coleman , J. C. Alvarez , S. D. Dreher , R. M. Garbaccio , N. K. Terrett , R. D. Tillyer , M. D. Truppo , E. R. Parmee , Science 2019, 363, 824; 10.1126/science.aat0805 30655413

[2d] N. Carson , Chem. Eur. J. 2020, 26, 3194–3196. 32125726 10.1002/chem.202000656

[3a] M. D. Burke , S. L. Schreiber , Angew. Chem. Int. Ed. 2004, 43, 46–58; 10.1002/anie.200300626 14694470

[3b] F. Lovering , J. Bikker , C. Humblet , J. Med. Chem. 2009, 52, 6752–6756; 19827778 10.1021/jm901241e

[3c] F. Lovering , MedChemComm 2013, 4, 515; Potential racemization should also be considered:

[3d] A. Ballard , H. O. Ahmad , S. Narduolo , L. Rosa , N. Chand , D. A. Cosgrove , P. Varkonyi , N. Asaad , S. Tomasi , N. J. Buurma , A. G. Leach , Angew. Chem. Int. Ed. 2018, 57, 982–985; 10.1002/anie.201709163 PMC5820753 29072355

[4a] T. J. Ritchie , S. J. Macdonald , Drug Discovery Today 2009, 14, 1011–1020; 19729075 10.1016/j.drudis.2009.07.014

[4b] A. D. Morley , A. Pugliese , K. Birchall , J. Bower , P. Brennan , N. Brown , T. Chapman , M. Drysdale , I. H. Gilbert , S. Hoelder , A. Jordan , S. V. Ley , A. Merritt , D. Miller , M. E. Swarbrick , P. G. Wyatt , Drug Discovery Today 2013, 18, 1221–1227; 23906694 10.1016/j.drudis.2013.07.011

[4c] N. A. Meanwell , Chem. Res. Toxicol. 2016, 29, 564–616. 26974882 10.1021/acs.chemrestox.6b00043

[5a] H. Caner , E. Groner , L. Levy , I. Agranat , Drug Discovery Today 2004, 9, 105–110; 15038394 10.1016/s1359-6446(03)02904-0

[5b] I. Agranat , S. R. Wainschtein , E. Z. Zusman , Nat. Rev. Drug Discovery 2012, 11, 972–973. 23197042 10.1038/nrd3657-c1

[6] A. Calcaterra , I. D′Acquarica , J. Pharm. Biomed. Anal. 2018, 147, 323–340. 28942107 10.1016/j.jpba.2017.07.008

[7a] J. M. Hawkins , T. J. Watson , Angew. Chem. Int. Ed. 2004, 43, 3224–3228; 10.1002/anie.200330072 15213946

[7b] H. J. Federsel , Nat. Mater. Nat. Rev. Drug. Discov. 2005, 4, 685–697. 10.1038/nrd1798 16041317

[8a] P. G. Cozzi , R. Hilgraf , N. Zimmermann , Eur. J. Org. Chem. 2007, 5969–5994;

[8b] C. Hawner , A. Alexakis , Chem. Commun. 2010, 46, 7295–7306; 10.1039/c0cc02309d 20734008

[8c] J. P. Das , I. Marek , Chem. Commun. 2011, 47, 4593–4623; 10.1039/c0cc05222a 21359322

[8d] K. W. Quasdorf , L. E. Overman , Nature 2014, 516, 181–191; 25503231 10.1038/nature14007 PMC4697831

[8e] D. Pierrot , I. Marek , Angew. Chem. Int. Ed. 2020, 59, 36–49; 10.1002/anie.201903188 31081180

[9] V. Farina , J. T. Reeves , C. H. Senanayake , J. J. Song , Chem. Rev. 2006, 106, 2734–2793. 16836298 10.1021/cr040700c

[10] A search on Scopus, using the combined terms “cataly*” and “asymmetric”, limited to primary research articles, letters, and notes in the Chemistry domain, found 33,941 entries in the period 1924–2019.

[11a] K. Tomioka , Y. Nagaoka in Comprehensive Asymmetric Catalysis, Vol. 3 (Eds.: Jacobsen E. N., Pfaltz A., Yamamoto H.), Springer-Verlag, Heidelberg, 1999;

[11b] E. M. Carreira , L. Kvaerno in Classics in Stereoselective Synthesis, Wiley-VCH, Weinheim, 2008.

[12] Following the broader citation search in reference [10], a search carried out by the authors on the combined terms “tertiary” OR “quaternary” AND “stereocentre” OR “stereocenter”, limited to primary research articles, letters, and notes in the Chemistry domain, found only 795 entries, with the first appearing in 1986.

[13] Discussion of related types of palladium relay transformations, such as intramolecular Heck–Matsuda reactions, which also occur by sequential migrations, are also out of scope for this article. For relevant examples, see:

[13a] J. G. Taylor , A. V. Moro , C. R. D. Correia , Eur. J. Org. Chem. 2011, 1403–1428;

[13b] R. C. Carmona , O. D. Koster , C. R. D. Correia , Angew. Chem. Int. Ed. 2018, 57, 12067–12070; 10.1002/anie.201805831 30020564

[14a] R. Delaby , C. R. Chim. 1926, 182, 140;

[14b] M. Kraus , Collect. Czech. Chem. Commun. 1972, 37, 460–465.

[15a] Z. Guan , P. M. Cotts , E. F. McCord , S. J. McLain , Science 1999, 283, 2059–2062; 10092223 10.1126/science.283.5410.2059

[15b] L. Guo , S. Dai , X. Sui , C. Chen , ACS Catal. 2016, 6, 428–441.

[16a] T. Hamasaki , Y. Aoyama , J. Kawasaki , F. Kakiuchi , T. Kochi , J. Am. Chem. Soc. 2015, 137, 16163–16171; 26633594 10.1021/jacs.5b10804

[16b] T. Hamasaki , F. Kakiuchi , T. Kochi , Chem. Lett. 2016, 45, 297–299;

[16c] A. Vasseur , J. Bruffaerts , I. Marek , Nat. Chem. 2016, 8, 209–219; 26892551 10.1038/nchem.2445

[16d] Y. Yamasaki , T. Kumagai , S. Kanno , F. Kakiuchi , T. Kochi , J. Org. Chem. 2018, 83, 9322–9333; 30022669 10.1021/acs.joc.8b01288

[16e] H. Sommer , F. Julia-Hernandez , R. Martin , I. Marek , ACS Cent. Sci. 2018, 4, 153–165; 29532015 10.1021/acscentsci.8b00005 PMC5833012

[16f] T. Kochi , K. Ichinose , M. Shigekane , T. Hamasaki , F. Kakiuchi , Angew. Chem. Int. Ed. 2019, 58, 5261–5265; 10.1002/anie.201814558 30748069

[17] R. Uma , C. Crévisy , R. Grée , Chem. Rev. 2003, 103, 27–52. 12517180 10.1021/cr0103165

[18a] E. Larionov , L. Lin , L. Guenee , C. Mazet , J. Am. Chem. Soc. 2014, 136, 16882–16894; 25397681 10.1021/ja508736u

[18b] L. Lin , C. Romano , C. Mazet , J. Am. Chem. Soc. 2016, 138, 10344–10350; 27434728 10.1021/jacs.6b06390

[18c] C. Romano , D. Fiorito , C. Mazet , J. Am. Chem. Soc. 2019, 141, 16983–16990. 31587562 10.1021/jacs.9b09373

[19a] J. L. Brooks , L. Xu , O. Wiest , D. S. Tan , J. Org. Chem. 2017, 82, 57–75; 28004933 10.1021/acs.joc.6b02053 PMC5224347

[19b] M. C. Lux , M. L. Boby , J. L. Brooks , D. S. Tan , Chem. Commun. 2019, 55, 7013–7016. 10.1039/c9cc03775f PMC6601619 31147660

[20] T. Kochi , T. Hamasaki , Y. Aoyama , J. Kawasaki , F. Kakiuchi , J. Am. Chem. Soc. 2012, 134, 16544–16547. 22998107 10.1021/ja308377u

[21a] J. T. Link in Organic Reactions, 2004, pp. 157–561;

[21b] R. F. Heck in Organic Reactions, 2005, pp. 345–390.

[22a] T. Mizoroki , K. Mori , A. Ozaki , Bull. Chem. Soc. Jpn. 1971, 44, 581;

[22b] R. F. Heck , J. P. Nolley , J. Org. Chem. 1972, 37, 2320–2322.

[23a] I. P. Beletskaya , A. V. Cheprakov , Chem. Rev. 2000, 100, 3009–3066; 11749313 10.1021/cr9903048

[23b] W. Cabri , I. Candiani , Acc.Chem. Res. 1995, 28, 2–7;

[23c] D. Mc Cartney , P. J. Guiry , Chem. Soc. Rev. 2011, 40, 5122–5150; 21677934 10.1039/c1cs15101k

[23d] J. Ruan , J. Xiao , Acc. Chem. Res. 2011, 44, 614–626; 21612205 10.1021/ar200053d

[23e] S. Jagtap , Catalysts 2017, 7, 267.

[24a] R. F. Heck , J. Am. Chem. Soc. 1968, 90, 5526–5531;

[24b] A. J. Chalk , S. A. Magennis , J. Org. Chem. 1976, 41, 1206–1209;

[24c] J. B. Melpolder , R. F. Heck , J. Org. Chem. 1976, 41, 265–272;

[24d] K. Von Werner , J. Organomet. Chem. 1977, 136, 385–387;

[24e] Y. Tamaru , Y. Yamada , Z. Yoshida , J. Org. Chem. 1978, 43, 3396–3398;

[24f] Y. Tamaru , Y. Yamada , Z.-I. Yoshida , Tetrahedron 1979, 35, 329–340;

[24g] S. Bouquillon , B. Ganchegui , B. Estrine , F. Hénin , J. Muzart , J. Organomet. Chem. 2001, 634, 153–156;

[24h] F. Berthiol , H. Doucet , M. Santelli , Tetrahedron 2006, 62, 4372–4383.

[25] A. J. Chalk , S. A. Magennis , J. Org. Chem. 1976, 41, 273–278.

[26a] C. M. Andersson , A. Hallberg , G. D. Daves , J. Org. Chem. 1987, 52, 3529–3536;

[26b] R. C. Larock , W. H. Gong , J. Org. Chem. 1989, 54, 2047–2050;

[26c] R. N. Farr , R. A. Outten , J. C. Y. Cheng , G. D. Daves , Organometallics 1990, 9, 3151–3156;

[26d] R. C. Larock , Y. D. Lu , A. C. Bain , C. E. Russell , J. Org. Chem. 1991, 56, 4589–4590;

[26e] R. C. Larock , N. G. Berrios-Pena , C. A. Fried , E. K. Yum , C. Tu , W. Leong , J. Org. Chem. 1993, 58, 4509–4510;

[26f] R. C. Larock , Y. Wang , Y. Lu , C. A. Russell , J. Org. Chem. 1994, 59, 8107–8114;

[26g] Y. Wang , X. Dong , R. C. Larock , J. Org. Chem. 2003, 68, 3090–3098; 12688777 10.1021/jo026716p

[26h] C. W. Holzapfel , D. B. G. Williams , Synth. Commun. 1994, 24, 2139–2146.

[27a] R. C. Larock , W.-Y. Leung , S. Stolz-Dunn , Tetrahedron Lett. 1989, 30, 6629–6632;

[27b] J. Masllorens , S. Bouquillon , A. Roglans , F. Hénin , J. Muzart , J. Organomet. Chem. 2005, 690, 3822–3826.

[28] W. Smadja , S. Czernecki , G. Ville , C. Georgoulis , Organometallics 1987, 6, 166–169.

[29] K. M. Gligorich , M. S. Sigman , Chem. Commun. 2009, 3854–3867. 10.1039/b902868d PMC2873851 19662234

[30a] E. W. Werner , M. S. Sigman , J. Am. Chem. Soc. 2010, 132, 13981–13983; 20858011 10.1021/ja1060998 PMC3011814

[30b] E. W. Werner , M. S. Sigman , J. Am. Chem. Soc. 2011, 133, 9692–9695. 21627305 10.1021/ja203164p PMC3124000

[31] E. W. Werner , T. S. Mei , A. J. Burckle , M. S. Sigman , Science 2012, 338, 1455–1458. 23239733 10.1126/science.1229208 PMC3583361

[32a] S. Yasui , M. Fujii , C. Kawano , Y. Nishimura , A. Ohno , Tetrahedron Lett. 1991, 32, 5601–5604;

[32b] W. Khodja , A. Leclair , J. Rull-Barrull , F. Zammattio , K. V. Kutonova , M. E. Trusova , F.-X. Felpin , M. Rodriguez-Zubiri , New. J. Chem. 2016, 40, 8855–8862.

[33] J.-Y. Guo , Y. Minko , C. B. Santiago , M. S. Sigman , ACS Catal. 2017, 7, 4144–4151.

[34] C. Zhang , C. B. Santiago , J. M. Crawford , M. S. Sigman , J. Am. Chem. Soc. 2015, 137, 15668–15671. 26624236 10.1021/jacs.5b11335 PMC5039010

[35a] C. C. Oliveira , R. A. Angnes , C. R. Correia , J. Org. Chem. 2013, 78, 4373–4385; 23570395 10.1021/jo400378g

[35b] C. C. Oliveira , A. Pfaltz , C. R. Correia , Angew. Chem. Int. Ed. 2015, 54, 14036–14039; 10.1002/anie.201507927 26404102

[36] D. Cortés-Borda , K. V. Kutonova , C. Jamet , M. E. Trusova , F. Zammattio , C. Truchet , M. Rodriguez-Zubiri , F.-X. Felpin , Org. Process Res. Dev. 2016, 20, 1979–1987.

[37a] J. Wilimowska , W. Piekoszewski , E. Krzyanowska-Kierepka , E. Florek , Clin. Toxicol. 2006, 44, 169–171; 10.1080/15563650500514541 16615674

[37b] V. Srinivasan , H. Sivaramakrishnan , B. Karthikeyan , Sci. Pharm. 2011, 79, 555–568. 21886903 10.3797/scipharm.1101-19 PMC3163365

[38] S. Kattela , E. C. de Lucca Jr , C. R. D. Correia , Chem. Eur. J. 2018, 24, 17691–17696. 30290051 10.1002/chem.201804958

[39] H. H. Patel , M. S. Sigman , J. Am. Chem. Soc. 2015, 137, 3462–3465. 25738548 10.1021/ja5130836 PMC4785804

[40] H. H. Patel , M. S. Sigman , J. Am. Chem. Soc. 2016, 138, 14226–14229. 27768842 10.1021/jacs.6b09649 PMC5152571

[41a] H. H. Patel , M. B. Prater , S. O. Squire Jr , M. S. Sigman , J. Am. Chem. Soc. 2018, 140, 5895–5898; 29665329 10.1021/jacs.8b02751 PMC5968819

[41b] M. B. Prater , M. S. Sigman , Isr. J. Chem. 2020, 60, 452–460. 33446940 10.1002/ijch.201900077 PMC7806183

[42] Z. M. Chen , J. Liu , J. Y. Guo , M. Loch , R. J. DeLuca , M. S. Sigman , Chem. Sci. 2019, 10, 7246–7250. 31588293 10.1039/c9sc02380a PMC6685350

[43] N. J. Race , Q. Yuan , M. S. Sigman , Chem. Eur. J. 2019, 25, 512–515. 30402891 10.1002/chem.201805416 PMC6342193

[44] Q. Yuan , M. S. Sigman , Chem. Eur. J. 2019, 25, 10823–10827. 31216370 10.1002/chem.201902813 PMC6731067

[45] T.-S. Mei , E. W. Werner , A. J. Burckle , M. S. Sigman , J. Am. Chem. Soc. 2013, 135, 6830–6833. 23607624 10.1021/ja402916z PMC3698857

[46a] X. Du , M. Suguro , K. Hirabayashi , A. Mori , T. Nishikata , N. Hagiwara , K. Kawata , T. Okeda , H. F. Wang , K. Fugami , M. Kosugi , Org. Lett. 2001, 3, 3313–3316; 11594822 10.1021/ol016529y

[46b] K. S. Yoo , C. H. Yoon , K. W. Jung , J. Am. Chem. Soc. 2006, 128, 16384–16393. 17165795 10.1021/ja063710z PMC2602842

[47] A. L. Lee , Org. Biomol. Chem. 2016, 14, 5357–5366. 26529247 10.1039/c5ob01984b

[48] C. Zhang , C. B. Santiago , L. Kou , M. S. Sigman , J. Am. Chem. Soc. 2015, 137, 7290–7293. 26030059 10.1021/jacs.5b04289 PMC4785797

[49] T.-S. Mei , H. H. Patel , M. S. Sigman , Nature 2014, 508, 340–344. 24717439 10.1038/nature13231 PMC4007023

[50] L. Xu , M. J. Hilton , X. Zhang , P.-O. Norrby , Y.-D. Wu , M. S. Sigman , O. Wiest , J. Am. Chem. Soc. 2014, 136, 1960–1967. 24410393 10.1021/ja4109616 PMC3985895

[51] J. Liu , Q. Yuan , F. D. Toste , M. S. Sigman , Nat. Chem. 2019, 11, 710–715. 31308495 10.1038/s41557-019-0289-7 PMC6679931

[52] H. Mei , J. Han , S. Fustero , M. Medio-Simon , D. M. Sedgwick , C. Santi , R. Ruzziconi , V. A. Soloshonok , Chem. Eur. J. 2019, 25, 11797–11819. 31099931 10.1002/chem.201901840

[53a] S. Singh , J. Bruffaerts , A. Vasseur , I. Marek , Nat. Commun. 2017, 8, 14200; 28169276 10.1038/ncomms14200 PMC5309700

[53b] S. Singh , M. Simaan , I. Marek , Chem. Eur. J. 2018, 24, 8553–8557. 29694690 10.1002/chem.201802016

[54] J. Bruffaerts , D. Pierrot , I. Marek , Nat. Chem. 2018, 10, 1164–1170. 30150723 10.1038/s41557-018-0123-7 PMC6197432

[55] J. R. Allen , A. Bahamonde , Y. Furukawa , M. S. Sigman , J. Am. Chem. Soc. 2019, 141, 8670–8674. 31117643 10.1021/jacs.9b01476 PMC6583780

[56] Q. Yuan , M. S. Sigman , J. Am. Chem. Soc. 2018, 140, 6527–6530. 29746119 10.1021/jacs.8b02752 PMC5993423

[57] Q. Yuan , M. B. Prater , M. S. Sigman , Adv. Synth. Catal. 2020, 362, 326–330. 33447174 10.1002/adsc.201901239 PMC7806184

[58a] Y. Dang , S. Qu , Z.-X. Wang , X. Wang , J. Am. Chem. Soc. 2014, 136, 986–998; 24380644 10.1021/ja410118m

[58b] M. J. Hilton , L.-P. Xu , P.-O. Norrby , Y.-D. Wu , O. Wiest , M. S. Sigman , J. Org. Chem. 2014, 79, 11841–11850. 25186804 10.1021/jo501813d PMC4275159

[59a] A. A. Sabino , A. H. Machado , C. R. Correia , M. N. Eberlin , Angew. Chem. Int. Ed. 2004, 43, 2514–2518; 10.1002/anie.200353076 15127439

[59b] J. C. Holder , L. Zou , A. N. Marziale , P. Liu , Y. Lan , M. Gatti , K. Kikushima , K. N. Houk , B. M. Stoltz , J. Am. Chem. Soc. 2013, 135, 14996–15007; 24028424 10.1021/ja401713g PMC3846424

[59c] A. H. Machado , H. M. Milagre , L. S. Eberlin , A. A. Sabino , C. R. Correia , M. N. Eberlin , Org. Biomol. Chem. 2013, 11, 3277–3281. 23511838 10.1039/c3ob40142a

[60] M. J. Hilton , B. Cheng , B. R. Buckley , L. Xu , O. Wiest , M. S. Sigman , Tetrahedron 2015, 71, 6513–6518. 26392640 10.1016/j.tet.2015.05.020 PMC4573459

[61] C. Frota , E. C. Polo , H. Esteves , C. R. D. Correia , J. Org. Chem. 2018, 83, 2198–2209. 29364666 10.1021/acs.joc.7b03098

[62] Z.-M. Chen , M. J. Hilton , M. S. Sigman , J. Am. Chem. Soc. 2016, 138, 11461–11464. 27571167 10.1021/jacs.6b06994 PMC5039009

[63] A. Milo , E. N. Bess , M. S. Sigman , Nature 2014, 507, 210–214. 24622199 10.1038/nature13019

[64] H. Nakamura , K. Yasui , Y. Kanda , P. S. Baran , J. Am. Chem. Soc. 2019, 141, 1494–1497. 30636411 10.1021/jacs.8b13697 PMC6353666

[65] N. Della Ca , M. Fontana , E. Motti , M. Catellani , Acc. Chem. Res. 2016, 49, 1389–1400. 27333299 10.1021/acs.accounts.6b00165

[66a] Z.-S. Liu , G. Qian , Q. Gao , P. Wang , H.-G. Cheng , Q. Wei , Q. Liu , Q. Zhou , ACS Catal. 2018, 8, 4783–4788;

[66b] Z.-S. Liu , G. Qian , Q. Gao , P. Wang , H.-G. Cheng , Y. Hua , Q. Zhou , Tetrahedron 2019, 75, 1774–1780.

[67] S. Gao , G. Qian , H. Tang , Z. Yang , Q. Zhou , ChemCatChem 2019, 11, 5762–5765.

[68] A. Rosales , S. P. Ross , P. Helquist , P. O. Norrby , M. S. Sigman , O. Wiest , J. Am. Chem. Soc. 2020, 142, 9700–9707. 32249569 10.1021/jacs.0c01979 PMC7304536

[69] M. Butters , D. Catterick , A. Craig , A. Curzons , D. Dale , A. Gillmore , S. P. Green , I. Marziano , J. P. Sherlock , W. White , Chem. Rev. 2006, 106, 3002–3027. 16836307 10.1021/cr050982w

[70] N. Oger , M. d'Halluin , E. Le Grognec , F.-X. Felpin , Org. Process Res. Dev. 2014, 18, 1786–1801.

[71] P. M. Osterberg , J. K. Niemeier , C. J. Welch , J. M. Hawkins , J. R. Martinelli , T. E. Johnson , T. W. Root , S. S. Stahl , Org. Process Res. Dev. 2015, 19, 1537–1543. 26622165 10.1021/op500328f PMC4655819

[72] C. Romano , C. Mazet , J. Am. Chem. Soc. 2018, 140, 4743–4750. 29561600 10.1021/jacs.8b02134

[73] For an example of interupted migration using homoallylic protected amines, see: S. P. Ross , A. A. Rahman , M. S. Sigman , J. Am. Chem. Soc. 2020, 142, 10516–10525. 32412759 10.1021/jacs.0c03589 PMC7376753

[74] A. Bahamonde , B. Al Rifaie , V. Martin-Heras , J. R. Allen , M. S. Sigman , J. Am. Chem. Soc. 2019, 141, 8708–8711. 31124676 10.1021/jacs.9b03438 PMC6583784

[75] R. C. Carmona , C. R. D. Correia , Adv. Synth. Catal. 2015, 357, 2639–2643.

[76] Z. M. Chen , C. S. Nervig , R. J. DeLuca , M. S. Sigman , Angew. Chem. Int. Ed. 2017, 56, 6651–6654; 10.1002/anie.201703089 PMC5528850 28467031

